# Preparation and Synthetic Application of Naproxen-Containing Diaryliodonium Salts

**DOI:** 10.3390/molecules26113240

**Published:** 2021-05-28

**Authors:** Jun Zhou, Zhiyuan Bao, Panpan Wu, Chao Chen

**Affiliations:** 1 School of Biotechnology and Health Sciences, International Healthcare Innovation Institute (Jiangmen), Wuyi University, Jiangmen 529000, China; zhoujunwyu@126.com; 2 Key Laboratory of Bioorganic Phosphorus Chemistry & Chemical Biology (Ministry of Education), Department of Chemistry, Tsinghua University, Beijing 100084, China; bzy18@mails.tsinghua.edu.cn; 3 State Key Laboratory of Elemento-Organic Chemistry, Nankai University, Tianjin 300071, China

**Keywords:** naproxen, naproxen methyl ester, diaryliodonium salts, Koser’s reagent, functionalization

## Abstract

The synthesis of naproxen-containing diaryliodonium salts has been realized from naproxen methyl ester and ArI(OH)OTs activated by trimethylsilyl trifluoromethanesulfonate (TMSOTf) in a solvent mixture comprising dichloromethane and 2,2,2-trifluoroethanol (TFE). Those iodonium salts have been successfully used in the functionalization of an aromatic ring of naproxen methyl ester, including fluorination, iodination, alkynylation, arylation, thiophenolation, and amination and esterification reactions. Moreover, further hydrolysis of the obtained 5-iodo-naproxen methyl ester afforded 5-iodo-naproxen.

## 1. Introduction

**Figure 1:**
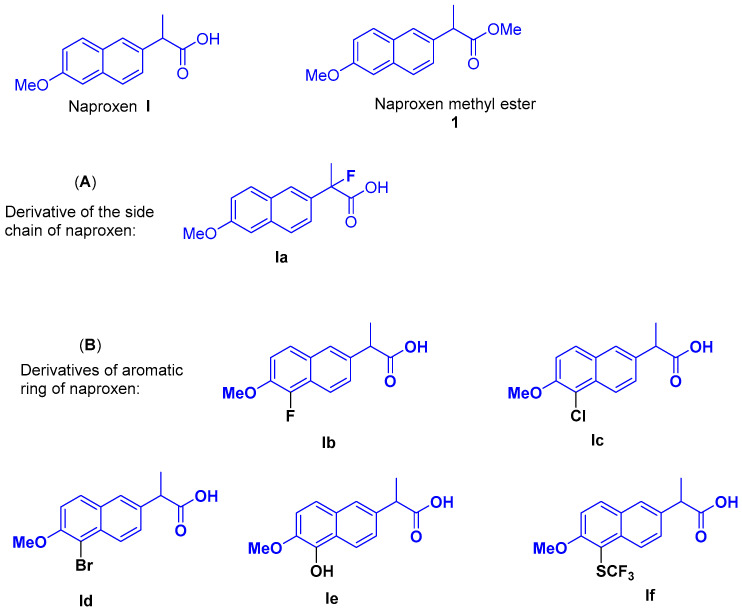
The structure of Naproxen and its typical derivatives: (**A**) the introduction of fluorine atom into the side chain; (**B**) the functionalization of the aromatic ring of naproxen.

Naproxen **I**, 2-(6-methoxynaphthalen-2-yl) propionic acid, is a widely prescribed nonsteroidal anti-inflammatory drug that relieves pain, fever, swelling, and stiffness. Naproxen is known to exert its protective effects as an anticancer agent [1]. Recently, naproxen has also been discovered to exhibit antiviral activity, reducing viral load in cells infected with influenza A (H1N1, H3N2) [2,3]. Industrial production of naproxen is currently about ten thousand tons per year, which creates a good basis for conducting research on its structural modification. Significant efforts have been made in the structural modification of naproxen [4,5], including the introduction of fluorine atom into the side chain of this molecule or an esterification process of the carboxylic acid with alcohols (Figure 1A, **Ia**) [6,7]. There have also been a few scattered methods for the functionalization of the aromatic ring of naproxen, including the introduction of the halogen atom into the aromatic ring (Figure 1B, **Ib**–**Id**) [8,9,10]. Some special functionalization has also been applied to the aromatic ring, including hydroxylation and the incorporation of the SCF_3_ group (Figure 1B, **Ie**, **If**) [11,12]. However, these separated methods and monotonous modifications are not conducive to the systematic expansion of naproxen. Therefore, there is an urgent and long-term need for more efficient and systematic methods to modify the aromatic ring of naproxen, particularly in the process of screening and discovering new drugs in medicinal chemistry. In this communication, we envision that the incorporation of the naproxen moiety with iodonium salts will open a new door to the generation of naproxen-based active molecules. Those naproxen-containing iodonium salts could serve as a versatile intermediate for library construction in medicinal chemistry.

**Scheme 1:**
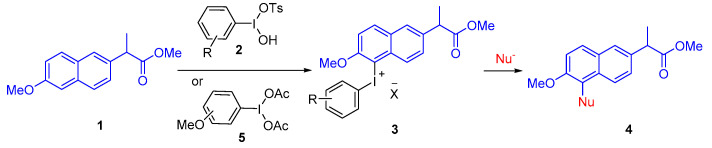
Preparation and synthetic application of naproxen methyl ester diaryliodonium salts.

In the past several decades, hypervalent iodine chemistry has witnessed prosperous development in organic synthesis [13,14,15]. Diaryliodonium salts, as one kind of the best-known iodine (III) compounds, are widely used as arylating agents and offer an alternative approach to realizing aryl group transformations under mild conditions. Stable iodonium salts have been found to have numerous practical applications, and a summary of the biological properties of iodonium salts is provided in review [14]. In a specific example, a study of the in vitro activities of several iodonium salts against oral and dental anaerobes has demonstrated that their activities are comparable to that of chlorhexidine and these compounds may be suitable for incorporation into an oral mouthwash [16]. Since the pioneering contributions of Kita et al., λ^3^-iodanes have been known to substitute electron-rich arenes with certain nucleophiles (e.g., N_3_, CN, OAc) [17,18,19,20,21,22,23]. As part of our long-term interest in the synthesis and application of hypervalent iodine compounds [24,25,26,27,28,29], we envision that diaryliodonium salts may serve the purpose of the modification of naproxen backbone. In this communication, we would like to report an effective approach to the synthesis of naproxen-containing diaryliodonium salts, i.e., naproxen methyl ester diaryliodonium salts, and the modification of the aromatic ring by substitution from formed naproxen methyl ester diaryliodonium salts (Scheme 1).

## 2. Results and Discussion

### 2.1. Selective Synthesis of Naproxen Methyl Ester Diaryliodonium Salts

**Scheme 2:**
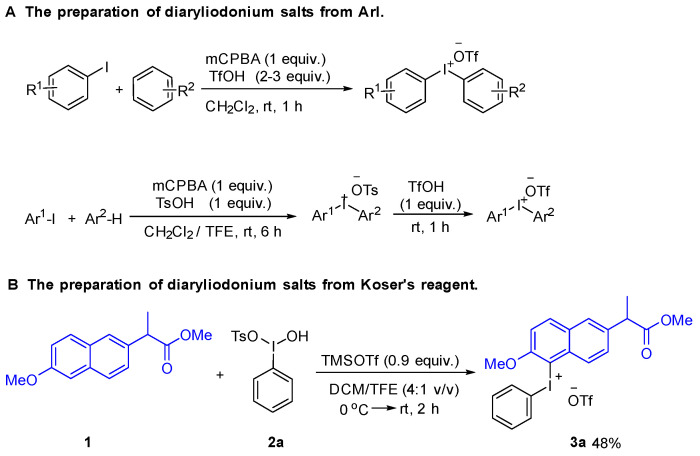
Some known methods for the preparation of diaryliodonium salts: (**A**) The preparation of diaryliodonium salts from ArI with mCPBA; (**B**) The preparation of diaryliodonium salts from the Koser’s reagent.

**Figure 2:**
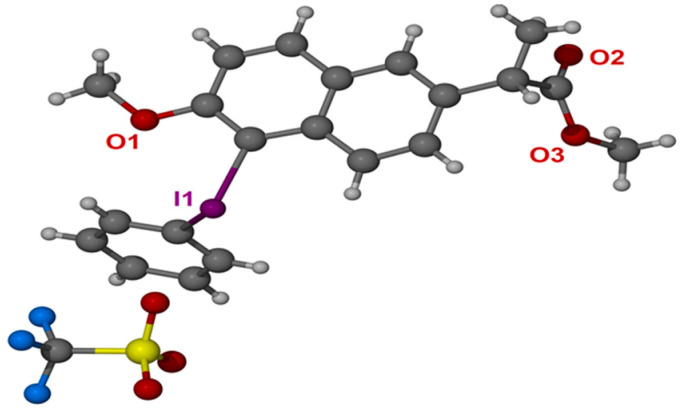
X-ray crystal structure of **3a** (CCDC no. 2050698).

At the outset of the study, we attempted to synthesize naproxen-containing iodonium salts according to the literature as shown in Scheme 2A [30,31]. Unfortunately, we did not get the desired iodonium salts by these methods. We speculated that the carboxylic acid on the side chain may interfere with the formation of diaryliodonium salts. Therefore, we decided to complete methylation of the carboxylic acid group. The methylation of naproxen with methanol and sulfuric acid was carried out at 70 °C. Koser’s reagent and its derivatives were prepared according to the reported literature [32]. The initial reactivity assay employed a simple Koser’s reagent **2a** as the model substrate to optimize the reaction parameters. Initially, the reaction conditions were optimized for naproxen methyl ester diaryliodonium salts with Koser’s reagent in dichloromethane [33]. However, the low solubility of Koser’s reagent in dichloromethane might prevent the formation of the diaryliodonium salt. As known, Koser’s reagent could be dissolved with TFE as a co-solvent. In this case, diaryliodonium salts were observed by TLC when one equivalent of TMSOTf was added, but it could not be crystalized from ether. To our surprise, when 0.9 equivalent of TMSOTf was added, naproxen methyl ester diaryliodonium salt was formed as a white solid from ether (Scheme 2B). Herein, TMSOTf was employed to activate the Koser’s reagent and simultaneously deliver the triflate anion to the salts. Furthermore, the structure of **3a** was undoubtedly confirmed by X-ray analysis (Figure 2; for details see Table S1 in the Supporting Information).

**Scheme 3:**
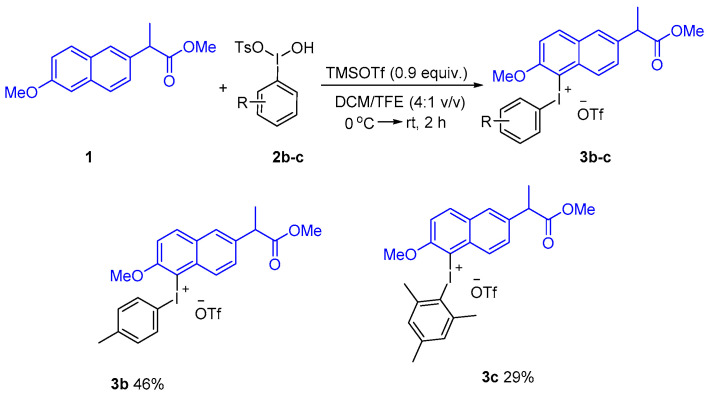
Preparation of naproxen methyl ester diaryliodonium salts from Koser’s reagent.

**Scheme 4:**
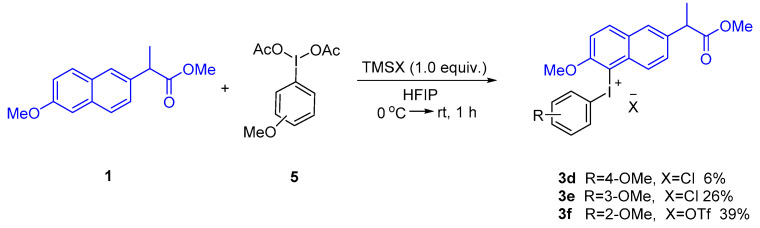
Preparation of naproxen methyl ester diaryliodonium salts from MeO-PhI(OAc)_2_.

Analogously, 4-methylphenyl and mesityl Koser’s reagents were successful in giving corresponding diaryliodonium salts by this approach (Scheme 3). However, in sharp contrast to the above method, the newly prepared 4-methoxy Koser’s reagent was quite unstable and decomposed violently during the isolation process [34]. After an extensive screening and optimization of reaction conditions, the corresponding 4-methoxyphenyl naproxen methyl ester diaryliodonium salt **3d** was prepared with 1-(diacetoxyiodo)-4-methoxybenzene activated by TMSOTf in a solvent of hexafluoroisopropanol at room temperature for 1 h in only 3% yield [35]. When TMSCl was used instead of TMSOTf, iodonium salt **3d** was obtained in 6% yield. The corresponding naproxen methyl ester diaryliodonium salt with 3-methoxyphenyl was prepared by the same method in 26% yield. Interestingly, TMSOTf was successfully used to prepare the corresponding naproxen methyl ester diaryliodonium salt with 2-methoxyphenyl in 39% yield (Scheme 4).

### 2.2. The Application of Naproxen Methyl Ester Diaryliodonium Salts

**Figure 3:**
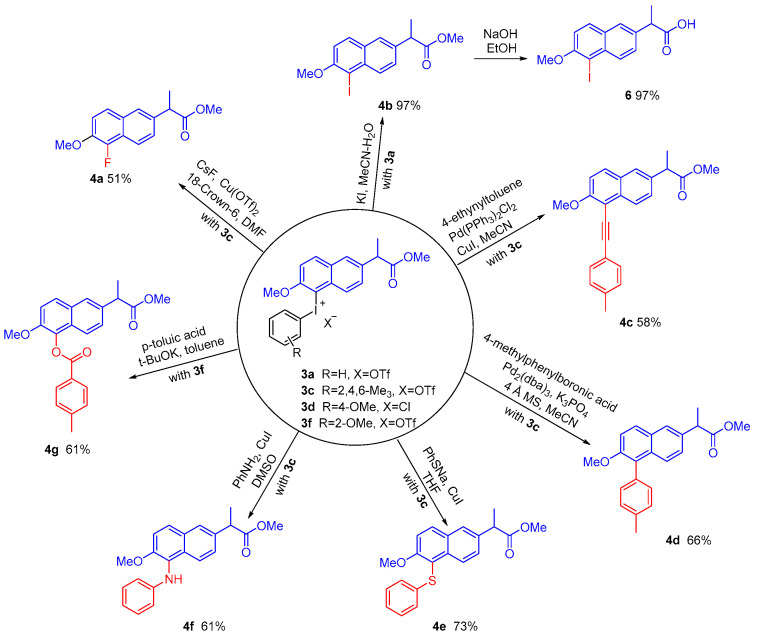
The modification of the aromatic ring of naproxen methyl ester from iodonium salts **3**.

Next, we attempted to investigate the synthetic application of these new naproxen methyl ester diaryliodonium salts. The mesityl naproxen methyl ester diaryliodonium salts **3c** reacted smoothly with CsF, 4-ethynyltoluene, 4-methylbenzeneboronic acid, sodium thiophenolate, and aniline to give substituted naproxen methyl ester in moderate to good yields (Figure 3, **4a**, **4c**–**4f**) [36,37,38]. However, when **3c** was treated with 4-methylbenzoic acid, the desired product was obtained in a low yield of 7% because of the preference to transfer the mesityl moiety. According to the general regular pattern and encouraged by Olofsson’s method, the methoxyphenyl group was then chosen as the dummy aromatic group of naproxen methyl ester diaryliodonium salts to improve selectivity [39,40]. As expected, the 4-methoxyphenyl naproxen methyl ester diaryliodonium salt **3d** reacted with 4-methylbenzoic acid to give aryl ester **4g** with good selectivity and yield (Figure 3, **4g**) and 2-methoxypheny iodonium salt **3f** showed the same good selectivity and yield as **3d**. In addition, iodonium salt **3a** reacted smoothly with KI to realize the iodination of naproxen methyl ester and further hydrolysis of the obtained 5-iodo naproxen methyl ester **4b** afforded 5-iodo-naproxen **6** in 97% yield (Figure 3, **4b** and **6**).

## 3. Materials and Methods

### 3.1. General Information

All reactions were carried out using a pre-dried screw capped tube with a Teflon-lined septum under nitrogen, unless otherwise noted. None of the solvents were dried before use, unless otherwise noted. The methylation of naproxen with methanol catalyzed by 10 mol% sulfuric acid was carried out at 70 °C. Koser’s reagent and its derivatives were prepared according to the reported literature [32], 1-(diacetoxyiodo)-4-methoxybenzene was prepared according to the reported literature [35], and other starting materials were commercially available and used without further purification. Column chromatography was performed using silica gel (particle size 10–40 μm, Ocean Chemical Factory of Qingdao, China). ^1^H-NMR, ^13^C-NMR, and ^19^F-NMR spectra were recorded on a JNM-ECA600, JNM-ECS400, or JNM-ECZ400S spectrometer (supplied by JEOL, Tokyo, Japan) at ambient temperature with CDCl_3_ or DMSO-*d_6_* as the solvent. Chemical shifts (δ) were given in ppm, referenced to the residual proton resonance of CDCl_3_ (7.26) or DMSO-*d_6_* (2.54), to the carbon resonance of CDCl_3_ (77.16) or DMSO-*d_6_* (40.45). Coupling constants (*J*) were given in Hertz (Hz). The terms m, q, t, d, and s refer to multiplet, quartet, triplet, doublet, and singlet, respectively. High-resolution mass spectra were acquired on LCMS-IT/TOF (Shimadzu, Kyoto, Japan) in an 80% acetonitrile−20% water mixture.

### 3.2. The Synthesis of Naproxen Methyl Ester Diaryliodonium Salts

**Method A for 3a–3c:** Naproxen methyl ester (1.22 g, 5.0 mmol) was added to a solution of ArI(OH)OTs (5.0 mmol) in DCM (15 mL) and 2,2,2-trifluoroethanol(TFE) (3.5 mL) in a 100 mL round-bottomed flask equipped with a stirring bar. The stirring solution was cooled to 0 °C and trimethylsilyl trifluoromethanesulfonate (TMSOTf) (815 µL, 4.5 mmol) was added dropwisely. The solution was warmed to room temperature and stirred for 2 h. The solvent was evaporated under reduced pressure, then ether (30 mL) was added. A white precipitate was separated, filtered with suction, and washed with ether (20 mL) to obtain the product as a white solid. If the diaryliodonium salt was not precipitated, ether was evaporated under reduced pressure and dried under vacuum for 1 h. Then, ether (30 mL) was added again and stirred vigorously. The white precipitate was separated, filtered with suction, and washed with ether (20 mL) to obtain the product as a white solid.

**Method B for 3d-3f:** Naproxen methyl ester (1.22 g, 5.0 mmol) was added to a solution of MeO-PhI(OAc)_2_ (5.0 mmol) in hexafluoroisopropanol (HFIP) (15 mL) in a 50 mL sealed tube. The stirring solution was cooled to 0 °C and trimethyl chlorosilane (TMSCl) (634 µL, 5.0 mmol) was added dropwisely. The solution was warmed to room temperature and stirred for 1 h. The solvent was evaporated under reduced pressure, then ether (30 mL) was added. A white precipitate was separated, filtered with suction, and washed with ether (5 mL) to obtain the product as a white solid.

*Naproxen methyl ester (phenyl) iodonium trifluoromethanesulfonate* (**3a**): White solid 1.43 g (2.4 mmol, 48%). M.p.: 126–127 °C.

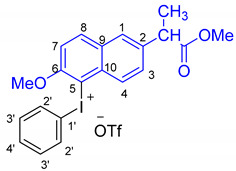



^1^H-NMR (600 MHz, CDCl_3_): δ 8.09 (d, *J* = 9.1 Hz, 1H, 8-H), 7.92 (d, *J* = 8.7 Hz, 1H, 4-H), 7.84 (d, *J* = 8.1 Hz, 2H, 2′-H), 7.74 (d, *J* = 1.3 Hz, 1H, 1-H), 7.60 (dd, *J* = 8.8, 1.6 Hz, 1H, 3-H), 7.45 (t, *J* = 7.4 Hz, 1H, 4′-H), 7.41 (d, *J* = 9.1 Hz, 1H, 7-H), 7.30 (t, *J* = 7.9 Hz, 2H, 3′-H), 4.05 (s, 3H, 6-OMe), 3.87 (q, *J* = 7.2 Hz, 1H, CH), 3.63 (s, 3H, COOMe), 1.54 (d, *J* = 7.2 Hz, 3H, CH_3_).

^13^C-NMR (151 MHz, CDCl_3_): δ 174.5(C=O), 157.6 (C-6), 138.1 (C-2), 137.1 (C-8), 134.3 (C-2′), 132.6 (C-10), 132.2 (C-4′), 132.1 (C-3′), 130.8 (C-3), 130.2 (C-9), 127.6 (C-1), 127.5 (C-4), 113.3 (C-7), 113.2 (C-1′), 101.2 (C-5), 58.0 (6-OMe), 52.3 (COOMe), 45.0 (CH), 18.4 (CH_3_).

^1^H,^1^H-COSY (600 MHz, CDCl_3_): δ ^1^H/^1^H = 8.09/7.41 (8-H/7-H), 7.92/7.60 (4-H/3-H), 7.84/7.30 (2′-H/3′-H), 7.30/7.45 (2′-H/3′-H), 3.87/1.54 (CH/CH_3_).

^1^H,^13^C-HMQC (600 MHz/151 MHz, CDCl_3_): δ ^1^H/^13^C = 8.09/137.1 (C-8), 7.92/127.5 (C-4), 7.84/134.3 (C-2′), 7.74/127.6 (C-1), 7.60/130.8 (C-3), 7.45/132.2 (C-4′), 7.41/113.3 (C-7), 7.30/132.1 (C-3′), 4.05/58.0 (6-OMe), 3.87/45.0 (CH), 3.63/52.3 (COOMe), 1.54/18.4 (CH_3_).

HRMS (ESI) calculated for [C_21_H_20_IO_3_]^+^ 447.0452 found: 447.0451. Element analysis: calculated for C_22_H_20_F_3_IO_6_S: C: 44.31%, H:3.38%; found: C: 44.36%, H: 3.34%.

The crystal suitable for XRD was obtained by the slow difussion of Et_2_O into its DCM solution. The crystal data are available via CCDC no. 2050698.

*Naproxen methyl ester (4-methylphenyl) iodonium trifluoromethanesulfonate* (**3b**): White solid 1.40 g (2.3 mmol, 46%). M.p.: 130–131 °C. ^1^H-NMR (400 MHz, CDCl_3_): δ 8.09 (d, *J* = 9.0 Hz, 1H), 7.95 (d, *J* = 8.7 Hz, 1H), 7.80–7.71 (m, 3H), 7.64 (d, *J* = 7.2 Hz, 1H), 7.39 (d, *J* = 9.1 Hz, 1H), 7.13 (d, *J* = 8.0 Hz, 2H), 4.09 (s, 3H), 3.90 (q, *J* = 7.2 Hz, 1H), 3.67 (s, 3H), 2.31 (s, 3H), 1.58 (d, *J* = 7.2 Hz, 3H). ^13^C-NMR (101 MHz, CDCl_3_): δ 174.5, 157.5, 143.3, 138.0, 137.0, 134.4, 132.9, 132.5, 130.8, 130.2, 127.6, 127.5, 113.3, 109.3, 101.2, 58.0, 52.3, 44.9, 21.3, 18.4. HR-MS (ESI) calculated for [C_22_H_22_IO_3_]^+^ 461.0608 found: 461.0609.

*Naproxen methyl ester (mesityl) iodonium trifluoromethanesulfonate* (**3c**): White solid 925 mg (1.45 mmol, 29%). M.p.: 160–161 °C.^1^H-NMR (400 MHz, CDCl_3_): δ 8.04 (d, *J* = 9.0 Hz, 1H), 7.97 (d, *J* = 8.7 Hz, 1H), 7.75 (s, 1H), 7.66 (d, *J* = 8.7 Hz, 1H), 7.29 (d, *J* = 9.0 Hz, 1H), 6.98 (s, 2H), 3.94 (s, 3H), 3.90 (q, *J* = 7.2 Hz, 1H), 3.68 (s, 2H), 2.57 (s, 6H), 2.27 (s, 3H), 1.58 (d, *J* = 7.2 Hz, 3H). ^13^C-NMR (101 MHz, CDCl_3_): δ 174.5, 157.6, 143.7, 142.5, 138.1, 136.5, 132.7, 130.8, 130.4, 130.2, 127.6, 127.2, 120.0, 113.4, 98.4, 57.6, 52.4, 45.0, 26.9, 21.0, 18.5. HR-MS (ESI) calculated for [C_24_H_26_IO_3_]^+^ 489.0921 found: 489.0924.

*Naproxen methyl ester (4-methoxyphenyl) iodonium chloride* (**3d**): White solid 153 mg (0.3 mmol, 6%). M.p.: 191–192 °C.^1^H-NMR (400 MHz, CDCl_3_): δ 8.09 (d, *J* = 9.0 Hz, 1H), 7.95 (d, *J* = 8.7 Hz, 1H), 7.82–7.70 (m, 3H), 7.64 (d, *J* = 8.3 Hz, 1H), 7.39 (d, *J* = 9.0 Hz, 1H), 7.14 (d, *J* = 8.3 Hz, 2H), 4.10 (s, 3H), 3.90 (q, *J* = 7.2 Hz, 1H), 3.67 (s, 3H), 2.31 (s, 3H), 1.58 (d, *J* = 7.2 Hz, 3H). ^13^C-NMR (101 MHz, CDCl_3_): δ 174.7, 161.3, 156.4, 137.3, 135.9, 134.9, 132.5, 130.1, 129.9, 128.6, 127.0, 116.8, 113.4, 110.9, 110.3, 57.9, 55.5, 52.3, 45.1, 18.6. HR-MS (ESI) calculated for [C_22_H_22_IO_4_]^+^ 477.0557 found: 477.0559.

*Naproxen methyl ester (3-methoxyphenyl) iodonium chloride* (**3e**): White solid 665 mg (1.3 mmol, 26%). M.p.: 164–165 °C. ^1^H-NMR (400 MHz, CDCl_3_): δ 8.15 (d, *J* = 8.7 Hz, 1H), 7.89 (d, *J* = 8.9 Hz, 1H), 7.63 (s, 2H), 7.54 (d, *J* = 8.8 Hz, 1H), 7.25 (dd, *J* = 8.5, 2.6 Hz, 2H), 7.00 (t, *J* = 8.1 Hz, 1H), 6.78 (dd, *J* = 8.2, 1.9 Hz, 1H), 3.97 (s, 3H), 3.82 (q, *J* = 7.0 Hz, 1H), 3.64 (s, 3H), 3.61 (s, 3H), 1.51 (d, *J* = 7.1 Hz, 3H). ^13^C-NMR (101 MHz, CDCl_3_): δ 174.6, 160.6, 156.8, 137.3, 135.1, 132.9, 131.3, 130.1, 129.9, 128.8, 126.9, 125.2, 119.9, 118.9, 116.7, 113.2, 109.4, 57.7, 55.7, 52.2, 45.0, 18.5. HR-MS (ESI) calculated for [C_22_H_22_IO_4_]^+^ 477.0557 found: 477.0558.

*Naproxen methyl ester (2-methoxyphenyl) iodonium trifluoromethanesulfonate* (**3f**): White solid 1.22 g (1.95 mmol, 39%). M.p.: 150–151 °C. ^1^H-NMR (400 MHz, CDCl_3_): δ 8.12 (d, *J* = 9.1 Hz, 1H), 7.91 (d, *J* = 8.8 Hz, 1H), 7.76 (d, *J* = 1.2 Hz, 1H), 7.62 (dd, *J* = 8.8, 1.6 Hz, 1H), 7.49–7.45 (m, 1H), 7.43 (d, *J* = 9.1 Hz, 1H), 7.29 (dd, *J* = 8.2, 1.2 Hz, 1H), 7.03 (dd, *J* = 8.3, 0.9 Hz, 1H), 6.91–6.87 (m, 1H), 4.06 (s, 3H), 3.91 (s, 4H), 3.67 (s, 3H), 1.58 (d, J = 7.2 Hz, 3H).^13^C-NMR (101 MHz, CDCl_3_): δ 174.6, 158.1, 156.3, 138.1, 137.2, 134.2, 133.5, 132.8, 130.6, 130.3, 128.1, 127.4, 124.2, 113.3, 112.8, 101.4, 98.7, 58.1, 57.1, 52.3, 45.0, 18.5. HR-MS (ESI) calculated for [C_22_H_22_IO_4_]^+^477.0557 found: 477.0558.

### 3.3. The Application of Naproxen Methyl Ester Diaryliodonium Salts

*Methyl 2-(5-fluoro-6-methoxynaphthalen-2-yl)propanoate* (**4a**)

Cu(OTf)_2_ (72 mg, 0.2 mmol), anhydrous C_S_F (91 mg, 0.6 mmol), and 18-crown-6 (26 mg, 0.1 mmol, 0.5 equivalent) were added to naproxen methyl ester (mesityl) iodonium trifluoromethanesulfonate **3c** (128 mg, 0.2 mmol) in a sealed tube. The reaction sealed tube was evacuated and backfilled with nitrogen 3 times, and anhydrous DMF (2 mL) was added via a syringe. The reaction mixture was stirred at 85 °C for 12 h. After cooling to room temperature, the reaction was quenched with saturated aqueous NaHCO_3_ (4 mL), and the mixture was diluted with DCM (2 × 10 mL) and washed with water (3 × 10 mL) and brine (2 × 10 mL). The organic phase was dried with anhydrous Na_2_SO_4_ and concentrated in vacuo. The crude material was purified by column chromatography on silica gel (PE/EtOAc = 15/1) to give **4a** (26 mg, 51% yield) as a white solid. M.p.: 53–54 °C. ^1^H-NMR (400 MHz, CDCl_3_): δ 8.10 (d, *J* = 8.7 Hz, 1H), 7.79 (d, *J* = 8.9 Hz, 1H), 7.64 (s, 1H), 7.49 (d, *J* = 7.6 Hz, 1H), 7.20 (d, *J* = 8.9 Hz, 1H), 4.01 (s, 3H), 3.89 (q, *J* = 7.2 Hz, 1H), 3.67 (s, 3H), 1.58 (d, *J* = 7.6, Hz, 3H). ^13^C-NMR (101 MHz, CDCl_3_): δ 175.0, 148.1, 145.6, 143.0, 142.9, 136.9, 126.7, 125.7, 123.9, 123.8, 120.3, 120.2, 57.8, 52.3, 45.5, 18.6. ^19^F-NMR (376 MHz, CDCl_3_) δ −146.96 (d, J = 8.1 Hz, 1F). HR-MS (ESI) calculated for C_15_H_15_FO_3_ [M + H]^+^ 263.1083 found: 263.1085.

*Methyl 2-(5-iodo-6-methoxynaphthalen-2-yl)propanoate* (**4b**)

*Naproxen methyl ester (phenyl) iodonium trifluoromethanesulfonate***3a** (119 mg, 0.2 mmol) and KI (166 mg, 1 mmol) were combined with MeCN (1.5 mL) and H_2_O (0.5 mL) in a 25 mL sealed tube. The reaction mixture was stirred at 100 °C for 12 h. After cooling to room temperature, the mixture was diluted with DCM (2 × 10 mL) and washed with water (10 mL) and brine (10 mL). The organic phase was dried with anhydrous Na_2_SO_4_ and concentrated in vacuo. The crude material was purified by column chromatography on silica gel (PE/EtOAc = 15/1) to give **4b** (71 mg, 97% yield) as a light yellow solid. M.p.: 57–58 °C. ^1^H-NMR (400 MHz, CDCl_3_): δ 8.11 (d, *J* = 8.8 Hz, 1H), 7.78 (d, *J* = 8.9 Hz, 1H), 7.65 (s, 1H), 7.49 (d, *J* = 8.8 Hz, 1H), 7.18 (d, *J* = 9.0 Hz, 1H), 4.00 (s, 3H), 3.89 (q, *J* = 7.2 Hz, 1H), 3.67 (s, 3H), 1.59 (d, *J* = 7.2 Hz, 3H). ^13^C-NMR (101 MHz, CDCl_3_): δ 175.0, 156.7, 136.6, 134.9, 131.8, 130.3, 129.9, 128.1, 126.4, 113.3, 87.5, 57.3, 52.2, 45.1, 18.6. HRMS (ESI) calculated for C_15_H_15_IO_3_ [M + H]^+^ 371.0144 found: 371.0141.

*Methyl 2-(6-methoxy-5-(p-tolylethynyl)naphthalen-2-yl)propanoate* (**4c**)

Pd(PPh_3_)_2_Cl_2_ (7 mg, 0.01 mol), CuI (7.6 mg, 0.04 mol), and Et_3_N (56 μL, 0.4 mmol) were added to naproxen methyl ester (mesityl) iodonium trifluoromethanesulfonate **3c** (128 mg, 0.2 mmol) in a sealed tube. The reaction sealed tube was evacuated and backfilled with nitrogen three times, and a solution of 4-Methylphenylacetylene (50 μL, 0.4 mmol) in anhydrous MeCN (2 mL) was added via a syringe. The reaction mixture was stirred at 50 °C under nitrogen atmosphere for 2 h. The reaction mixture was then diluted with DCM (2 × 10 mL), washed with water (10 mL) and brine (10 mL), dried over anhydrous Na_2_SO_4_, and concentrated under vacuo. The crude material was purified by column chromatography on silica gel (PE/EtOAc = 15/1) to give **4c** (41 mg, 58% yield) as a brownish yellow solid. M.p.: 50–51 °C.^1^H-NMR (400 MHz, DMSO-*d_6_*): δ 8.23 (d, *J* = 8.7 Hz, 1H), 8.02 (d, *J* = 9.1 Hz,1H), 7.85 (s,1H), 7.70–7.41 (m, 4H), 7.31 (d, *J* = 8.0 Hz, 2H), 4.04 (s, 3H), 3.99 (q, *J* = 7.2 Hz, 1H), 3.64 (s, 3H), 3.40 (s, 3H), 2.40 (s, 3H), 1.53 (d, *J* = 7.1 Hz, 3H). ^13^C-NMR (101 MHz, DMSO-*d_6_*): δ 175.2, 159.6, 139.3, 137.4, 133.7, 132.1, 131.3, 130.4, 129.0, 128.5, 127.3, 125.9, 120.9, 114.5, 105.7, 99.5, 84.4, 57.4, 52.8, 45.2, 22.0, 19.3. HR-MS (ESI) calculated for C_24_H_22_O_3_ [M + H]^+^ 359.1647 found: 359.1644.

*Methyl 2-(6-methoxy-5-(p-tolyl)naphthalen-2-yl)propanoate* (**4d**)

Pd_2_(dba)_3_ (18 mg, 0.02 mol), 4Å MS (100 mg), K_3_PO_4_(127 mg, 0.6 mmol), and 4-methylphenyl boronic acid (54 mg, 0.4 mmol) were added to naproxen methyl ester (mesityl) iodonium trifluoromethanesulfonate **3c** (128 mg, 0.2 mmol) in a sealed tube. The reaction sealed tube was evacuated and backfilled with nitrogen 3 times, anhydrous MeCN (2 mL) was added via a syringe. The reaction mixture was stirred at room temperature under nitrogen atmosphere for 12 h. The reaction mixture was then diluted with DCM (10 mL × 2), washed with water (10 mL) and brine (10 mL), dried over anhydrous Na_2_SO_4_, and concentrated under vacuum. The crude material was purified by column chromatography on silica gel (PE/EtOAc = 15/1) to give **4d** (44 mg, 66% yield) as a light-yellow oil. ^1^H-NMR (400 MHz, CDCl_3_): δ 7.83 (d, *J* = 9.0 Hz, 1H), 7.71 (d, *J* = 1.7 Hz, 1H), 7.49 (d, *J* = 8.8 Hz, 1H), 7.36 (d, *J* = 9.0 Hz, 1H), 7.31–7.22 (m, 5H), 3.86 (q, *J* = 7.2 Hz, 1H), 3.83 (s, 3H), 3.65 (s, 3H), 2.45 (s, 3H), 1.56 (d, *J* = 7.1 Hz, 3H). ^13^C-NMR (101 MHz, CDCl_3_): δ 175.2, 153.9, 136.8, 135.6, 133.3, 133.0, 130.8, 129.1, 129.0, 128.9, 126.2, 126.1, 126.0, 125.4, 114.2, 56.9, 52.2, 45.3, 21.5, 18.6. HR-MS (ESI) calculated for C_22_H_22_O_3_ [M + H]^+^ 335.1647 found: 335.1645.

*Methyl 2-(6-methoxy-5-(phenylthio)naphthalen-2-yl)propanoate* (**4e**)

Sodium thiophenolate (48 mg, 0.36 mol) and CuI (3 mg, 0.016 mmol) were added to naproxen methyl ester (mesityl) iodonium trifluoromethanesulfonate **3c** (77 mg, 0.12 mmol) in a sealed tube. The reaction sealed tube was evacuated and backfilled with nitrogen 3 times, anhydrous THF (1 mL) was added via a syringe. The reaction mixture was stirred at 40 °C under nitrogen atmosphere for 3 h. The reaction mixture was then diluted with DCM (10 mL × 2), washed with water (5 mL) and brine (5 mL), dried over anhydrous Na_2_SO_4_, and concentrated under vacuum. The crude material was purified by column chromatography on silica gel (PE/EtOAc = 25/1) to give **4f** (31 mg, 73% yield) as a colorless oil. ^1^H-NMR (400 MHz, CDCl_3_): δ 8.42 (d, *J* = 8.8 Hz, 1H), 7.94 (d, *J* = 9.1 Hz, 1H), 7.72 (d, *J* = 1.6 Hz, 1H), 7.45 (dd, *J* = 8.9, 1.9 Hz, 1H), 7.36 (d, *J* = 9.1 Hz, 1H), 7.15–7.11 (m, 2H), 7.06–7.01(m, 3H), 3.95 (s, 3H), 3.87 (q, *J* = 7.2 Hz, 1H), 3.67 (s, 3H), 1.58 (d, *J* = 7.1 Hz, 3H). ^13^C-NMR (101 MHz, CDCl_3_): δ 175.1, 159.4, 138.2, 136.4, 135.7, 132.1, 129.7, 128.8, 127.9, 126.5, 126.5, 126.1, 124.9, 113.9, 113.1, 57.1, 52.3, 45.3, 18.7. HR-MS (ESI) calculated for C_21_H_20_O_3_S [M + H]^+^ 353.1211 found: 353.1214.

*Methyl 2-(6-methoxy-5-(phenylamino)naphthalen-2-yl)propanoate* (**4f**)

A flame-dried sealed tube was charged with CuI (3 mg, 0.015 mmol) and naproxen methyl ester (mesityl) iodonium trifluoromethanesulfonate **3c** (77 mg, 0.12 mmol). The sealed tube was kept under a positive pressure of nitrogen. Anhydrous dimethyl sulfoxide (1 mL) and aniline (33 µL, 0.36 mol) were introduced via syringe. The reaction mixture was stirred at 140 °C under nitrogen atmosphere for 1 h. The reaction mixture was then diluted with DCM (10 mL × 2), washed with water (3 × 10 mL) and brine (10 mL), dried over anhydrous Na_2_SO_4_, and concentrated under vacuum. The crude material was purified by column chromatography on silica gel (PE/EtOAc = 30/1) to give **4g** (25 mg, 61% yield) as a colorless oil. ^1^H-NMR (400 MHz, CDCl_3_): δ 7.80 (d, J = 8.8 Hz, 1H), 7.71–7.68 (m, 2H), 7.33 (d, *J* = 9.0 Hz, 2H), 7.15 (dd, *J* = 8.2, 7.6 Hz, 2H), 6.81 (t, *J* = 7.3 Hz, 1H), 6.67 (d, *J* = 8.3 Hz, 2H), 3.91 (s, 3H), 3.86 (q, *J* = 7.2 Hz, 1H), 3.68 (s, 3H), 1.58 (d, *J* = 7.2 Hz, 3H). ^13^C-NMR (101 MHz, CDCl_3_): δ 175.2, 150.9, 146.9, 136.0, 129.9, 129.6, 129.1, 126.3, 126.1, 124.3, 124.3, 119.6, 115.7, 114.0, 56.8, 52.2, 45.4, 18.7. HR-MS (ESI) calculated for C_21_H_21_NO_3_ [M + H]^+^ 336.1600 found: 336.1603.

*2-Methoxy-6-(1-methoxy-1-oxopropan-2-yl)naphthalen-1-yl 4-methylbenzoate* (**4g**)

A flame-dried sealed tube was charged with a magnetic stir bar and tBuOK (31 mg, 0.28 mmol). The sealed tube was kept under a positive pressure of nitrogen. Anhydrous toluene (4 mL) was introduced via syringe, and 4-methylbenzoic acid (38 mg, 0.28 mmol) and naproxen methyl ester (4-methoxyphenyl) iodonium chloride **3d** (143 mg, 0.25 mmol) were added sequentially as solids. The sealed tube was then heated to reflux for 3 h. The reaction mixture was then diluted with DCM (10 mL × 2), washed with water (10 mL) and brine (10 mL), dried over anhydrous Na_2_SO_4_, and concentrated under vacuum. The crude material was purified by column chromatography on silica gel (PE/EtOAc = 15/1) to give **4g** (60 mg, 64% yield) as a colorless liquid. When 4-methylbenzoic acid was treated with naproxen methyl ester (2-methoxyphenyl) iodonium trifluoromethanesulfonate **3f**, the yield of **4g** was 61%. ^1^H-NMR (400 MHz, CDCl_3_): δ 8.24 (d, *J* = 8.2 Hz, 2H), 7.82–7.73 (m, 3H), 7.43–7.36 (m, 4H), 3.93 (s, 3H), 3.87 (q, *J* = 7.1 Hz, 1H), 3.66 (s, 1H), 2.49 (s, 3H), 1.58 (d, *J* = 7.2 Hz, 3H). ^13^C-NMR (101 MHz, CDCl_3_): δ 175.1, 164.8, 148.1, 144.6, 136.4, 133.6, 130.6, 129.5, 129.4, 127.6, 127.0, 126.8, 126.6, 126.2, 121.0, 114.7, 57.0, 52.2, 45.4, 22.0, 18.6. HR-MS (ESI) calculated for C_23_H_22_O_5_ [M + H]^+^ 379.1545 found: 379.1546.

*2-(5-Iodo-6-methoxynaphthalen-2-yl)propanoic acid* (**6**)

A 25 mL sealed tube was charged with 5-iodo naproxen methyl ester **4b** (56 mg, 0.15 mmol) and ethanol (0.5 mL). Then, a solution of sodium hydroxide (12 mg, 0.3 mmol) in ethanol (0.6 mL) was added slowly over 1 min. The mixture was stirred at reflux for about 3 h. After cooling the mixture to 0 °C, the pH value was adjusted to 2 by adding 1 N hydrogen dchloride. A standard extractive workup with ethyl acetate (3 × 5 mL) gave the product **6** (52 mg, 97% yield) as a white solid. M.p.: 177–178 °C. ^1^H-NMR (400 MHz, DMSO-*d_6_*): δ 12.43 (s, 1H), 8.02–7.99 (m, 2H), 7.81 (s, 1H), 7.57 (d, *J* = 7.2 Hz, 1H), 7.44 (d, *J* = 9.0 Hz, 1H), 4.00 (s, 3H), 3.89 (q, *J* = 7.1 Hz, 1H), 1.50 (d, *J* = 7.1 Hz, 3H). ^13^C-NMR (101 MHz, DMSO-*d_6_*): δ 176.3, 157.3, 138.0, 134.9, 131.5, 131.3, 130.3, 129.4, 127.3, 114.7, 87.6, 58.1, 45.3, 19.3. HR-MS (ESI) calculated for C_14_H_13_IO_3_ [M + H]^+^ 356.9988 found: 356.9987.

^1^H-NMR, ^13^C-NMR and ^19^F-NMR spectrum of compounds **3**, **4** and **6** are presented in Supplementary Material (Figures S2–S32).

## 4. Conclusions

In summary, naproxen-cantaining diaryliodonium salts were successfully synthesized from naproxen methyl ester and ArI(OH)OTs in dichloromethane and 2,2,2-trifluoroethanol. The structure of the iodonium salt **3a** was confirmed by X-ray diffraction. Moreover, those iodonium salts were successfully used in further derivatization, including fluorination, iodination, alkynylation, arylation, thiophenolation, and amination and esterification reactions. This strategy offered a new source for the generation of naproxen-based active molecules.

## Data Availability

The data presented in this study are available in the communication.

## Supplementary Materials

Supplementary Materials are available online. ^1^H-NMR,^13^C-NMR, and ^19^F-NMR spectroscopic data for all new compounds are presented in Figures S2–S32. Figure S1. X-Ray crystallographic structure for compound **3a**, Table S1. Details of data collection, processing and structure refinement for compound **3a**.

## Sample Availability

Samples of the compounds are not available from the authors.
