# Supplementary material for: Preparation and Synthetic Application of Naproxen-Containing Diaryliodonium Salts
Source: Molecules. 2021 May 28;26(11):3240. doi: 10.3390/molecules26113240 (PMC8198133; doi:10.3390/molecules26113240)
Supplement: Supplementary file 1 [file molecules-26-03240-s001.zip › molecules-1185272-supplementary.pdf]

molecules

Supplementary Material

Preparation and Synthetic Application of Naproxen-containing Diaryliodonium Salts

Jun Zhou [1], Zhiyuan Bao [2] and Panpan Wu [1,*], Chao Chen [2,3,*]

1 School of Biotechnology and Health Sciences, Wuyi University, Jiangmen, 529020, China & International Healthcare Innovation Institute (Jiangmen), Jiangmen, 529000, China
2 Key Laboratory of Bioorganic Phosphorus Chemistry & Chemical Biology (Ministry of Education), Department of Chemistry, Tsinghua University, Beijing 100084, China
3 State Key Laboratory of Elemento-Organic Chemistry, Nankai University, Tianjin 300071, China

Table of Contents

*1. X-Ray crystallographic Data(**3a**)* *3*
Figure S1. X-Ray crystallographic structure for compound **3a**........ *3*
Table S1. Details of Data Collection, Processing and Structure Refinement for Compound **3a***3*
2. ^{1}H-NMR , ^{13}C-NMR and ^{19}F-NMR Spectrum of All New Compounds *5*
Figure S2. ^{1}H-NMR Spectrum (600 MHz, $CDCl_3$) of **3a** 5
Figure S3 ^{13}C-NMR Spectrum (151 MHz, $CDCl_3$) of **3a**........ 5
Figure S4. ^{1}H,^{1}H-COSY Spectrum (600 MHz, $CDCl_3$) of **3a** 6
Figure S5. ^{1}H,^{13}C-HMQC Spectrum (600 MHz / 151 MHz, $CDCl_3$) of **3a**........ 7
Figure S6. ^{1}H-NMR Spectrum (400 MHz, $CDCl_3$) of **3b** 8
Figure S7. ^{13}C-NMR Spectrum (101 MHz, $CDCl_3$) of **3b** 8
Figure S8. ^{1}H-NMR Spectrum (400 MHz, $CDCl_3$) of **3c**........ 9
Figure S9. ^{13}C-NMR Spectrum (101 MHz, $CDCl_3$) of **3c**........ 9
Figure S10. ^{1}H-NMR Spectrum (400 MHz, $CDCl_3$) of **3d**........ 10
Figure S11. ^{13}C-NMR Spectrum (101 MHz, $CDCl_3$) of **3d** 10
Figure S13. ^{1}H-NMR Spectrum (400 MHz, $CDCl_3$) of **3e** 11
Figure S13. ^{1}H-NMR Spectrum (400 MHz, $CDCl_3$) of **3e** 11
Figure S14. ^{1}H NMR Spectrum (400 MHz, $CDCl_3$) of **3f** 12
Figure S15. ^{13}C-NMR Spectrum (101 MHz, $CDCl_3$) of **3f** 12
Figure S16. ^{1}H-NMR Spectrum (400 MHz, $CDCl_3$) of **4a** 13
Figure S17. ^{13}C-NMR Spectrum (101 MHz, $CDCl_3$) of **4a**........ 13
Figure S18. ^{19}F-NMR Spectrum (376 MHz, $CDCl_3$) of **4a** 14
Figure S19. ^{1}H-NMR Spectrum (400 MHz, $CDCl_3$) of **4b**........ 14
Figure S20. ^{13}C-NMR Spectrum (101 MHz, $CDCl_3$) of **4b** 15
Figure S21. ^{1}H-NMR Spectrum (400 MHz, DMSO-d6) of **4c**........ 15
Figure S22. ^{13}C-NMR Spectrum (101 MHz, DMSO-d6) of **4c** 16

Figure S23. ^{1}H-NMR Spectrum (400 MHz, $CDCl_3$) of **4d** 16

Figure S24. ^{13}C-NMR Spectrum (101 MHz, $CDCl_3$) of **4d** 17

Figure S25. ^{1}H-NMR Spectrum (400 MHz, $CDCl_3$) of **4e** 17

Figure S26. ^{13}C-NMR Spectrum (101 MHz, $CDCl_3$) of **4e** 18

Figure S27. ^{1}H-NMR Spectrum (400 MHz, CDCl3) of **4f** 18

Figure S28. ^{13}C-NMR Spectrum (101 MHz, $CDCl_3$) of **4f** 19

Figure S29. ^{1}H-NMR Spectrum (400 MHz, $CDCl_3$) of **4g** 19

Figure S30. ^{13}C-NMR Spectrum (101 MHz, $CDCl_3$) of **4g** 20

Figure S31. ^{1}H-NMR Spectrum (400 MHz, DMSO-d6) of **6** 20

Figure S32. ^{13}C-NMR Spectrum (101 MHz, DMSO-d6) of **6** 21

1. X-Ray crystallographic data (3a)

The crystal suitable for XRD was obtained by slow diffusion of Et_2O into its DCM solution. The crystal data is available via CCDC no. 2050698

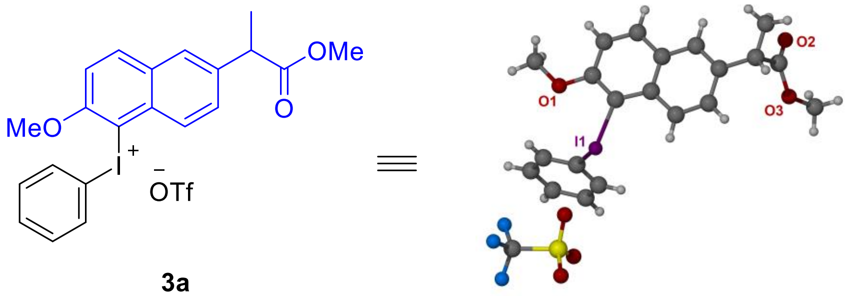

Figure S1. X-Ray crystallographic structure for compound **3a.**

Table S1. Details of Data Collection, Processing and Structure Refinement for Compound **3a.**

Bond precision:	C-C = 0.0102 A	
Wavelength	1.54184	
Cell:	a = 7.1638(3)	α = 68.683(5)
	b = 11.9805(5)	β = 82.539(4)
	c = 14.5345(9)	γ = 88.307(4)
Temperature:	173 K	
	Calculated	Reported
Volume	1152.04(11)	1152.04(11)
Space group	P -1	P -1
Hall group	-P 1	-P 1
Moiety formula	$C_{19}H_{17}IO$, CF_3O_3S, $C_2H_3O_2$	$C_{21}H_{20}IO_3$, CF_3O_3S
Sum formula	$C_{22}H_{20}F_3IO_6S$	$C_{22}H_{20}F_3IO_6S$
Mr	596.34	596.34
Dx, g cm^{-3}	1.719	1.719
Z	2	2

Mu (mm^{-1})	12.322	12.322
F000	592.0	592.0
F000′	594.00	
h, k, lmax	8, 14, 17	8, 14, 17
Nref	3926	3852
Tmin, Tmax	0.262, 0.292	0.554, 1.000
Tmin′	0.074	
Correction method = # Reported T Limits:	Tmin = 0.554	Tmax = 1.000
Data completeness = 0.981	Theta(max) = 65.051	
R(reflections) = 0.0465(3408)	wR2(reflections) = 0.1171(3852)	
S = 1.090	Npar = 338	

2. ^{1}H-NMR, ^{13}C-NMR and ^{19}F-NMR Spectrum of All New Compounds:

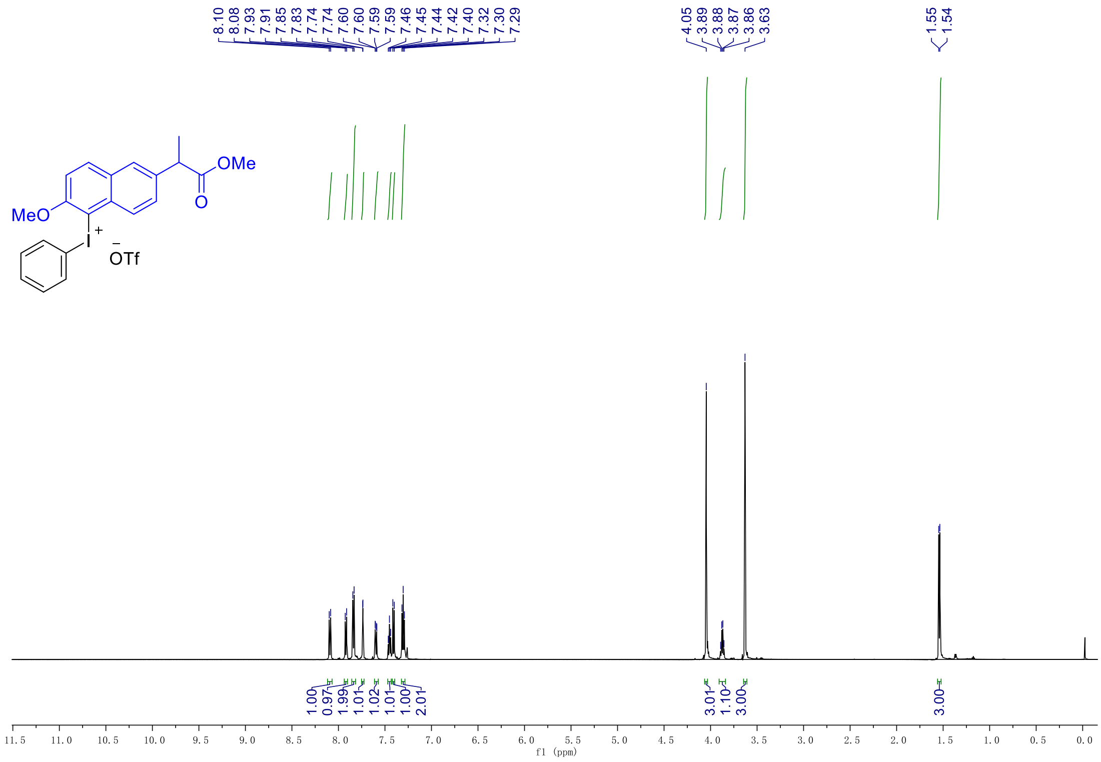

Figure S2. ^{1}H-NMR Spectrum (600 MHz, CDCl3) of **3a.**

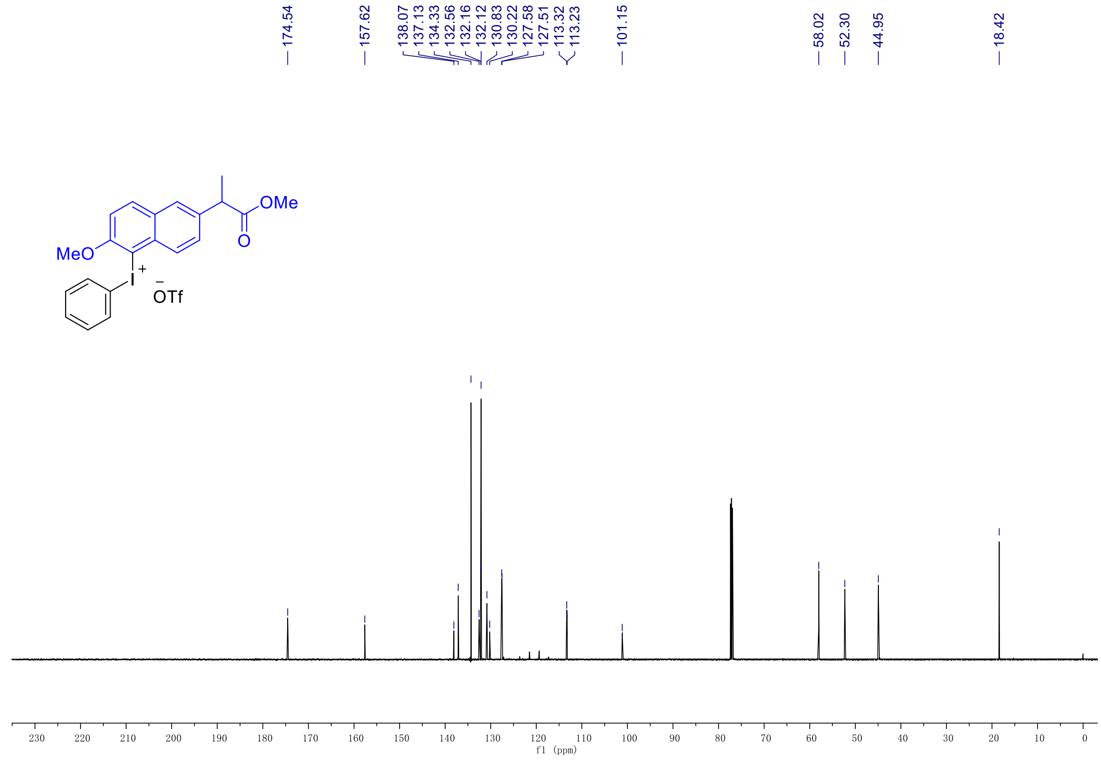

Figure S3. ^{13}C-NMR Spectrum (151 MHz, $CDCl_3$) of 3a.

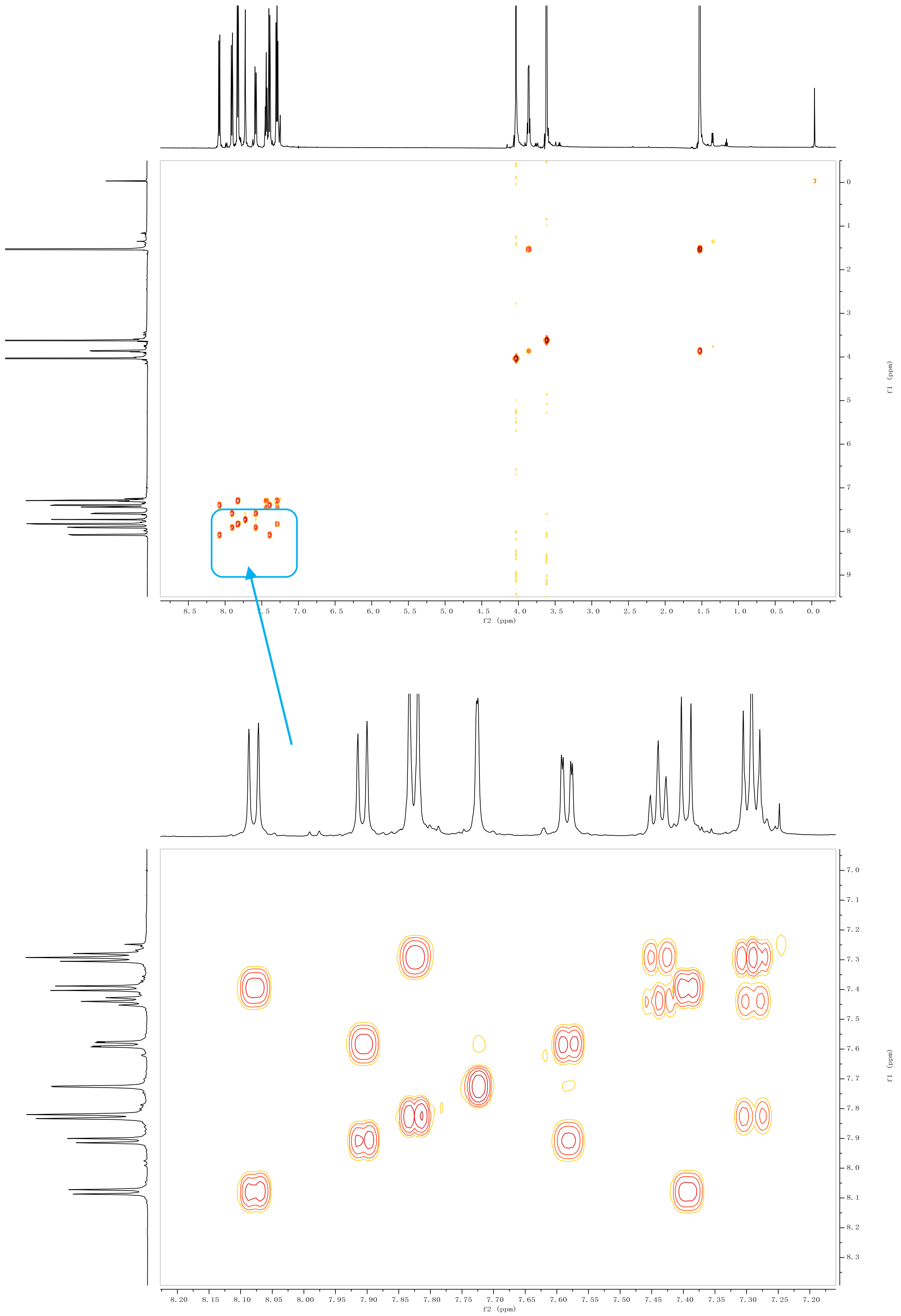

Figure S4. $^1H,^1H$-COSY Spectrum (600 MHz, $CDCl_3$) of **3a.**

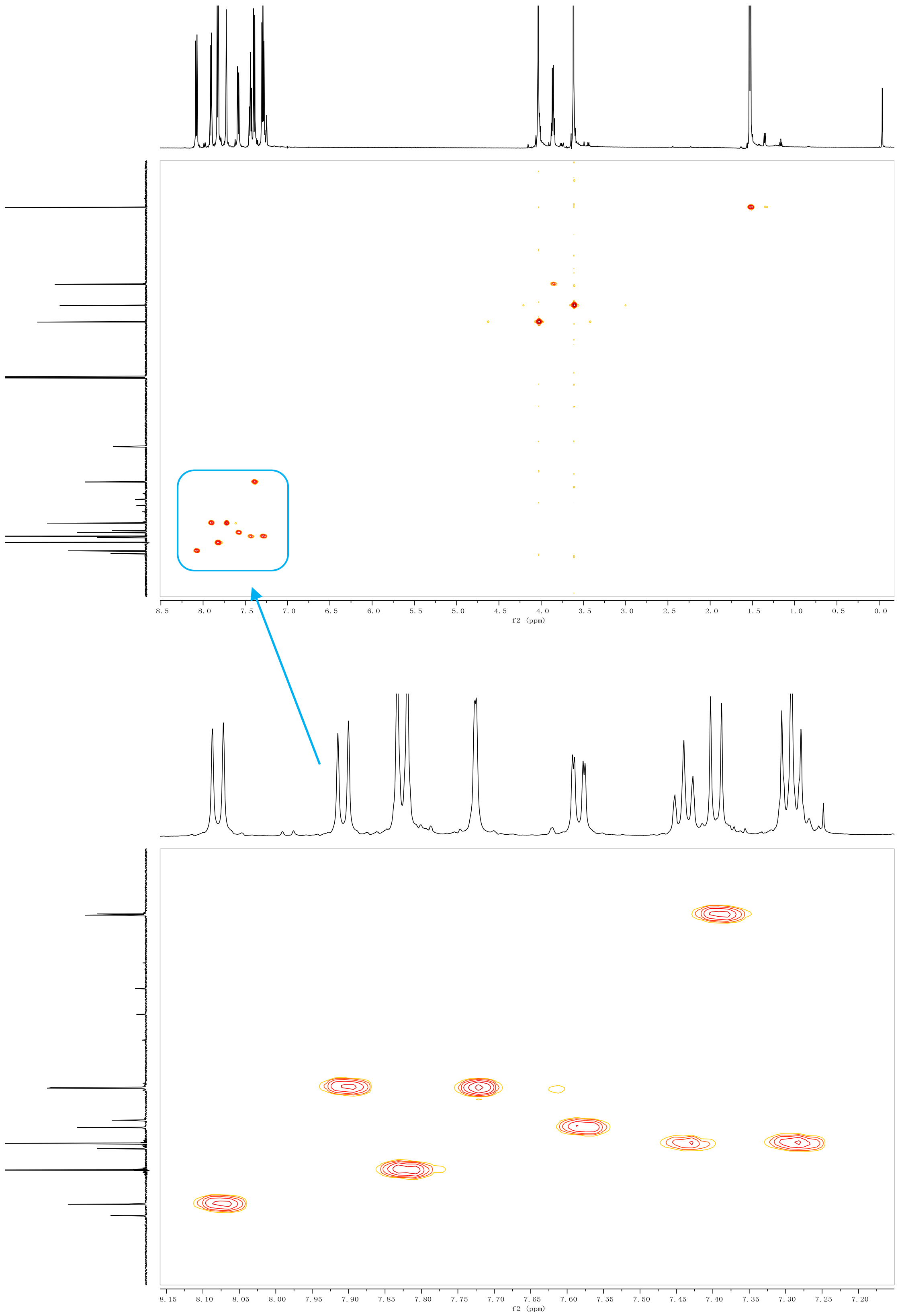

Figure S5. H,^{13}C-HMQC Spectrum (600 MHz / 151 MHz, $CDCl_3$) of **3a.**

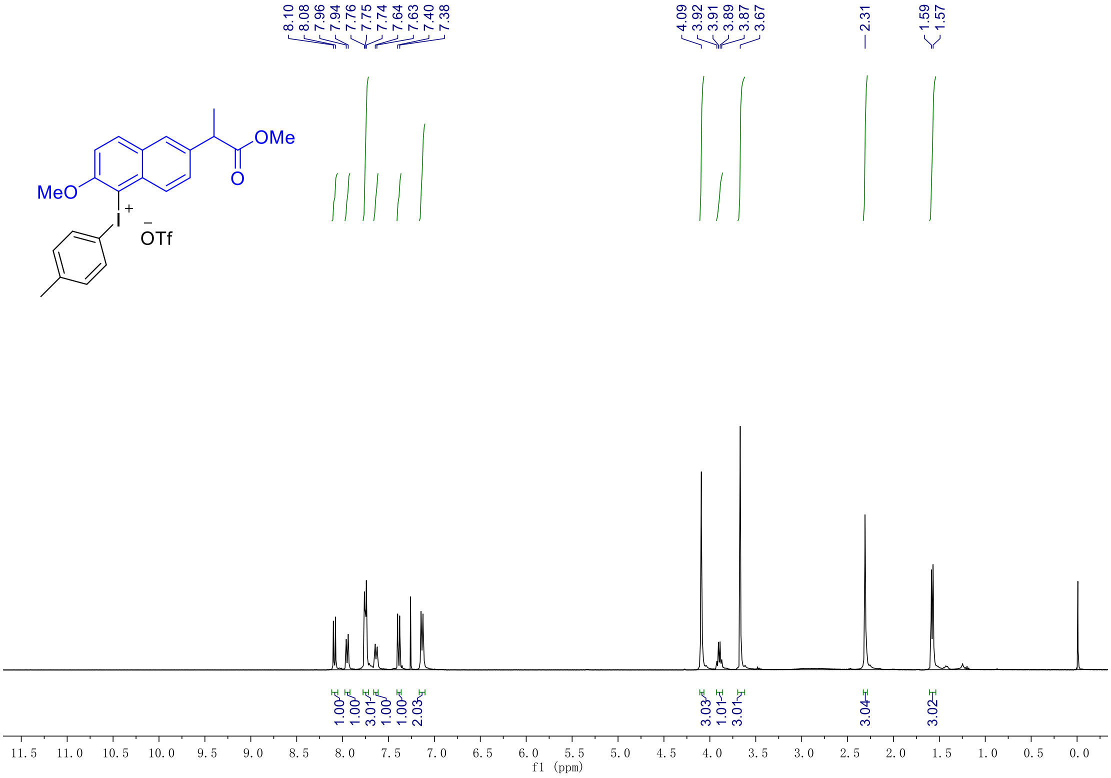

Figure S6. ^{1}H-NMR Spectrum (400 MHz, $CDCl_3$) of **3b.**

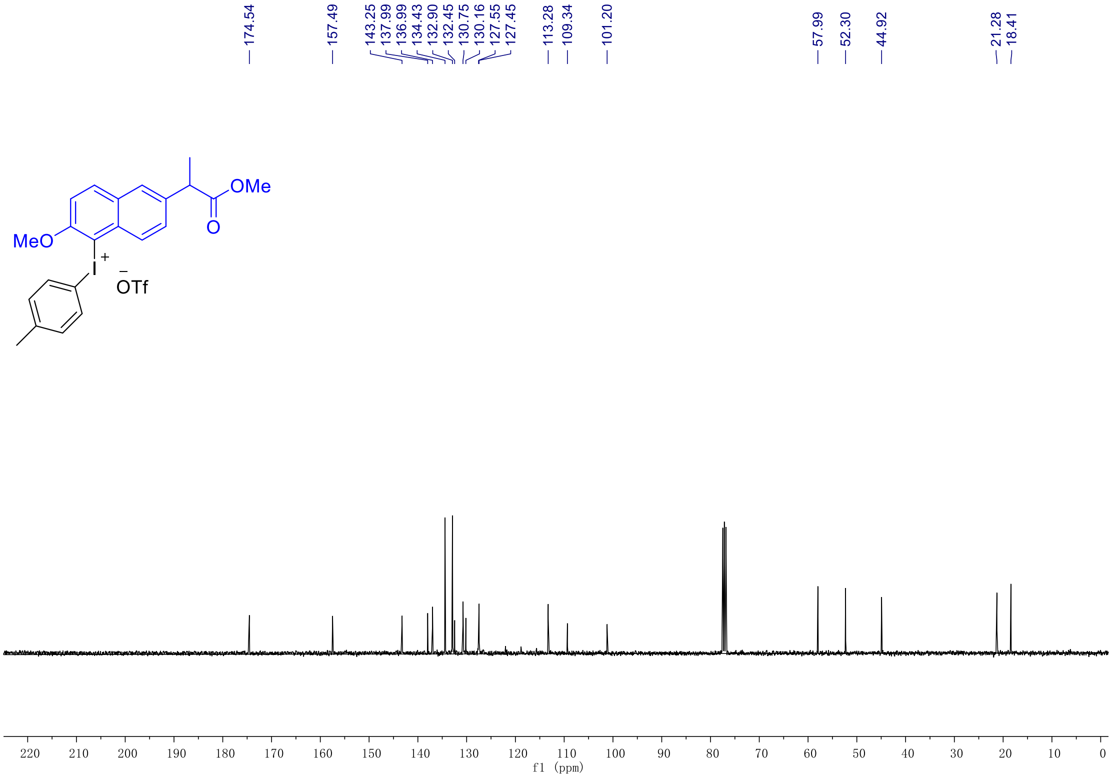

Figure S7. ^{13}C-NMR Spectrum (101 MHz, $CDCl_3$) of **3b.**

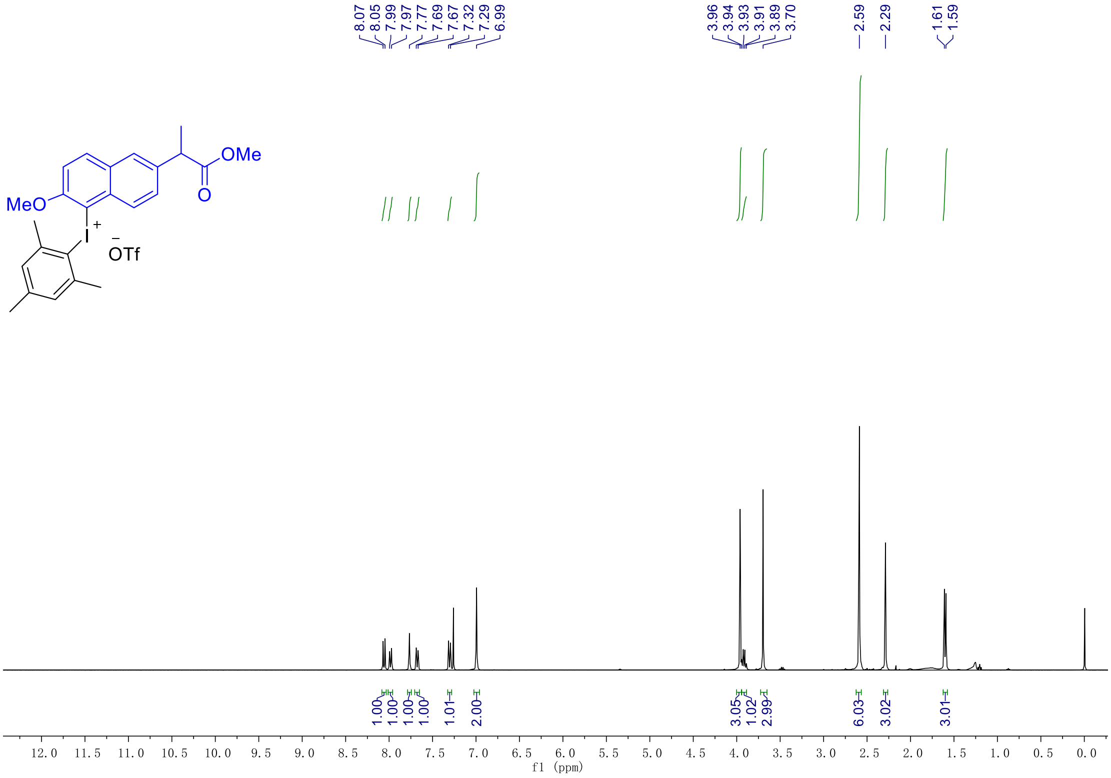

Figure S8. ^{1}H-NMR Spectrum (400 MHz, $CDCl_3$) of **3c.**

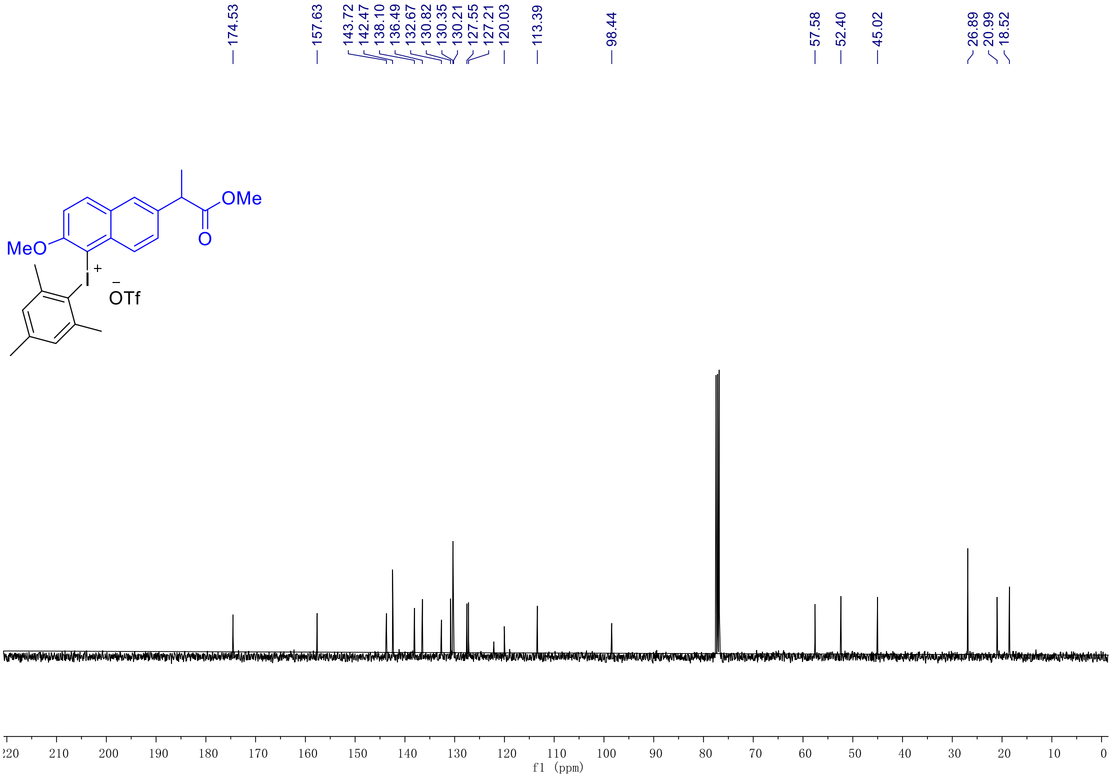

Figure S9. ^{13}C-NMR Spectrum (101 MHz, $CDCl_3$) of **3c.**

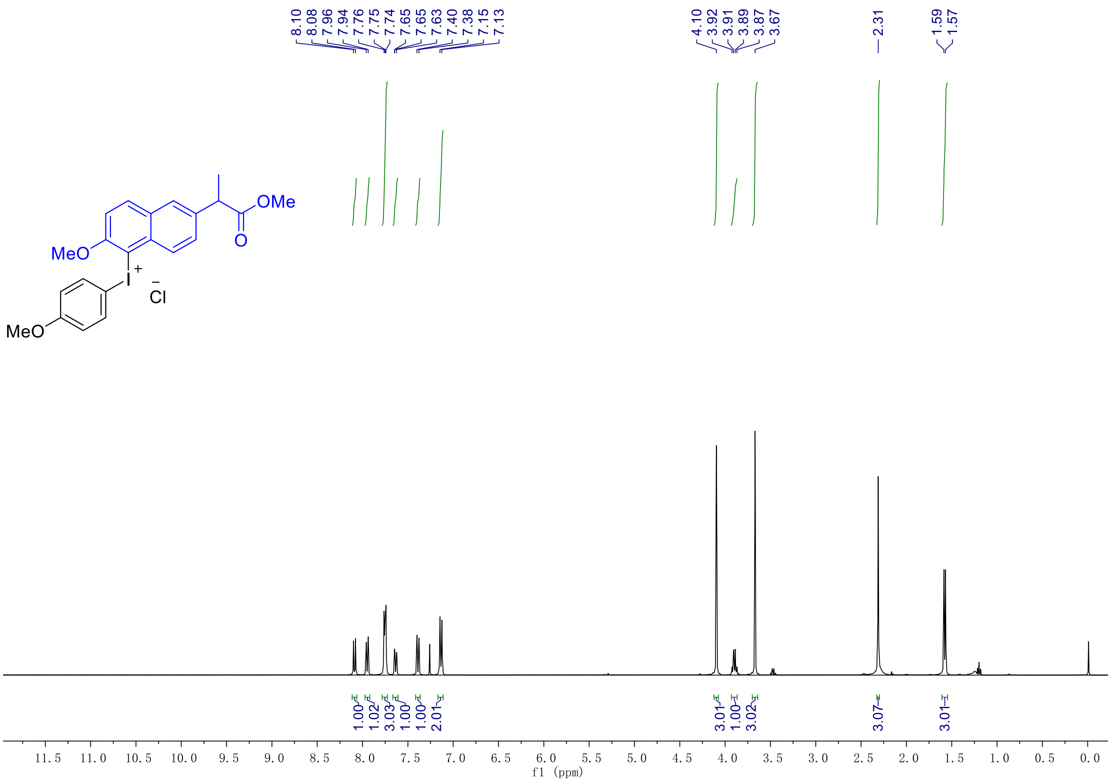

Figure S10. ^{1}H-NMR Spectrum (400 MHz, $CDCl_3$) of **3d.**

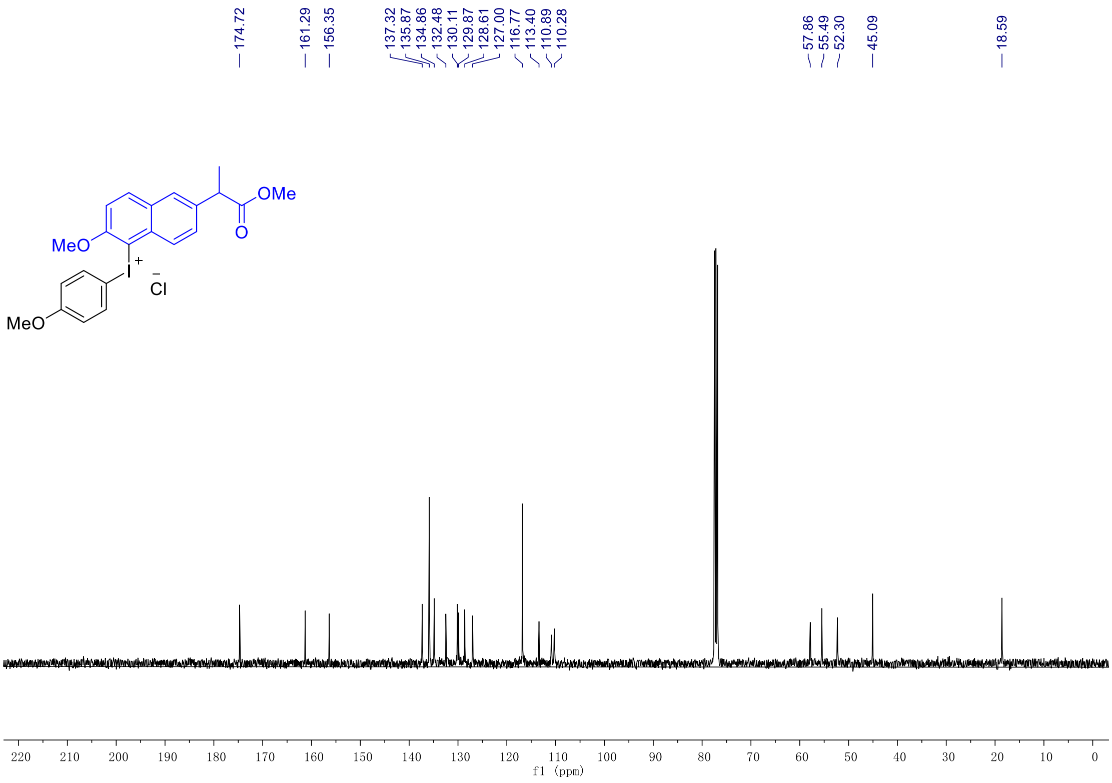

Figure S11. ^{13}C-NMR Spectrum (101 MHz, $CDCl_3$) of **3d.**

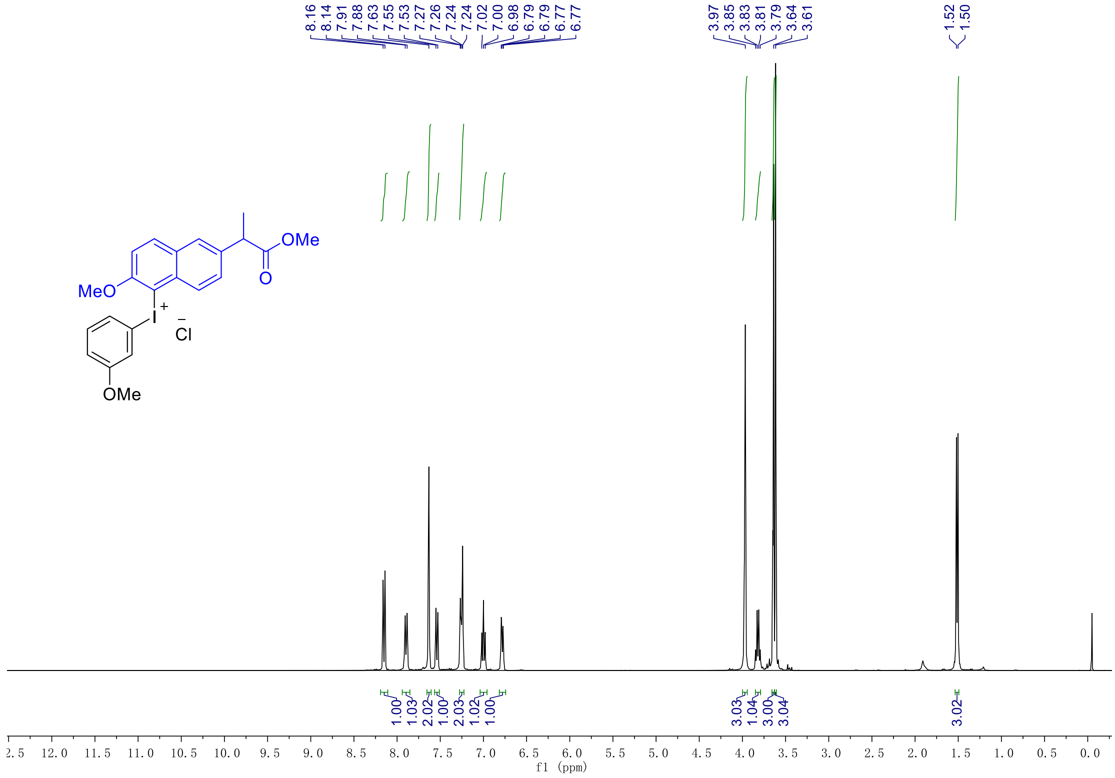

Figure S12. ^{1}H-NMR Spectrum (400 MHz, $CDCl_3$) of **3e.**

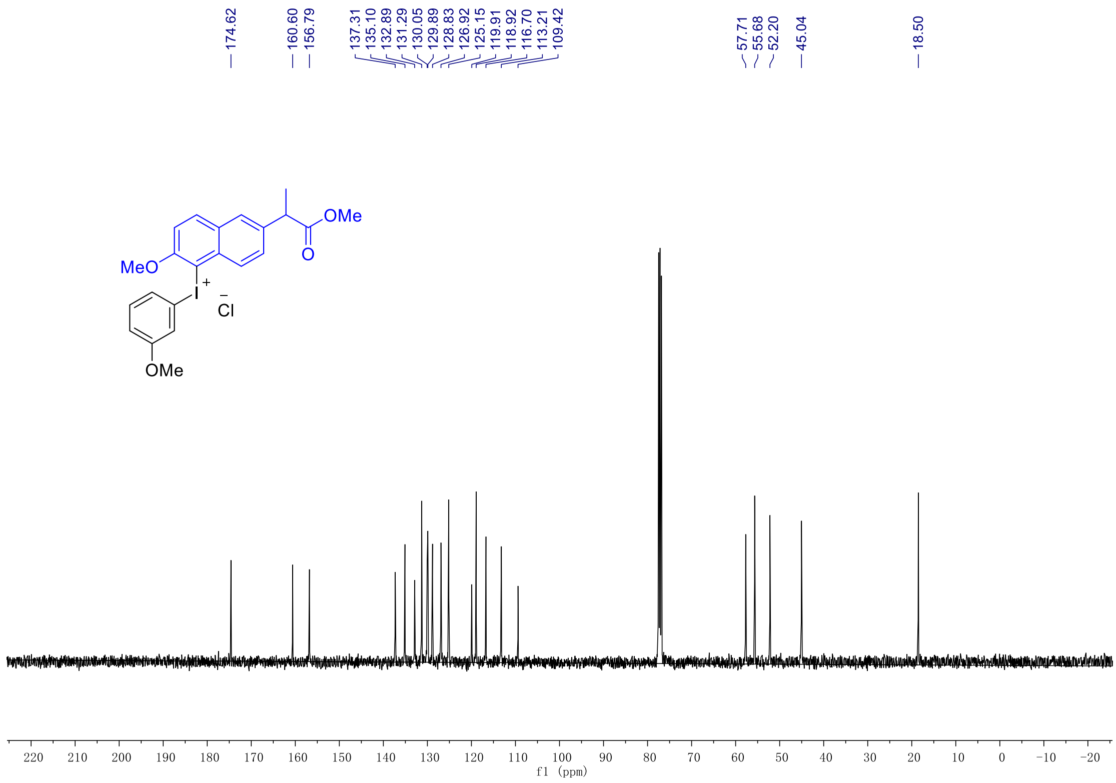

Figure S13. ^{1}H-NMR Spectrum (400 MHz, $CDCl_3$) of **3e.**

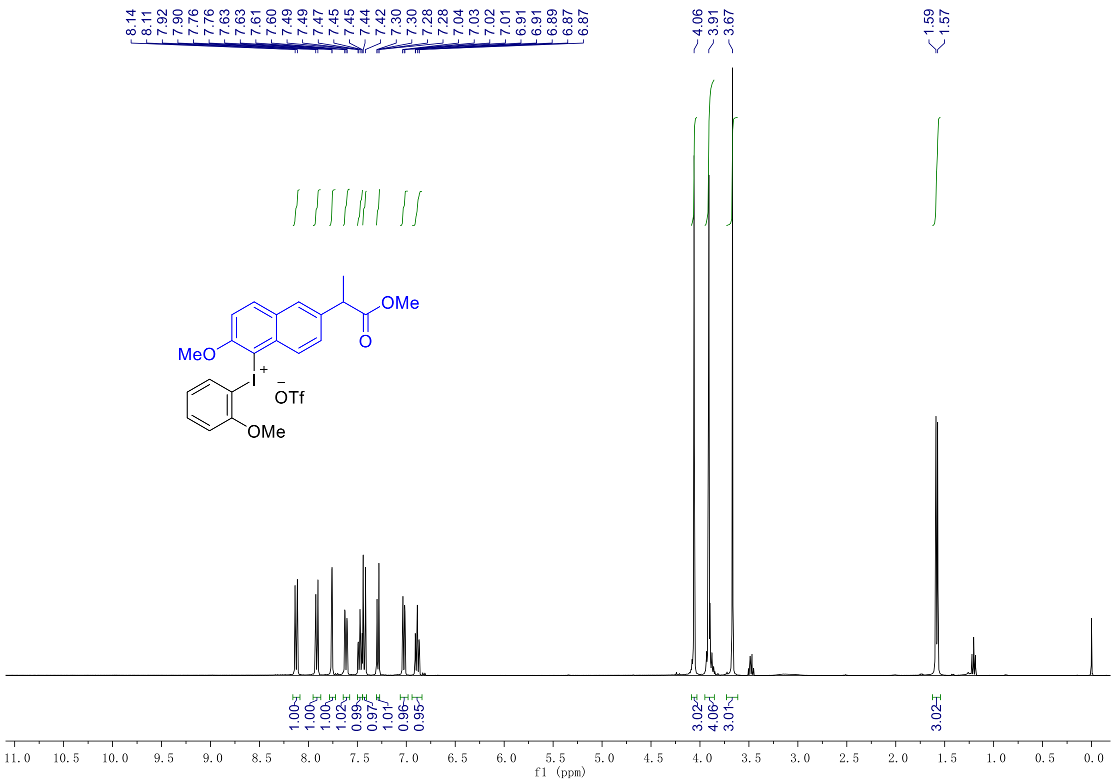

Figure S14. ^{1}H-NMR Spectrum (400 MHz, $CDCl_3$) of **3f.**

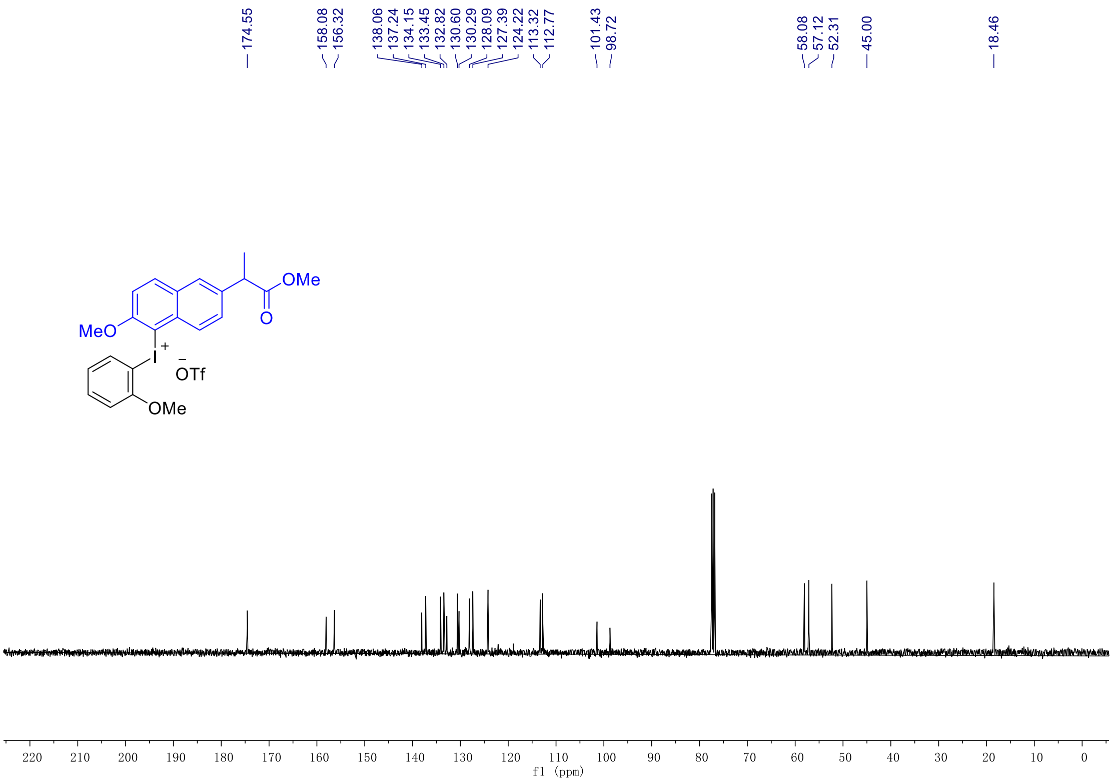

Figure S15. ^{13}C-NMR Spectrum (101 MHz, $CDCl_3$) of **3f.**

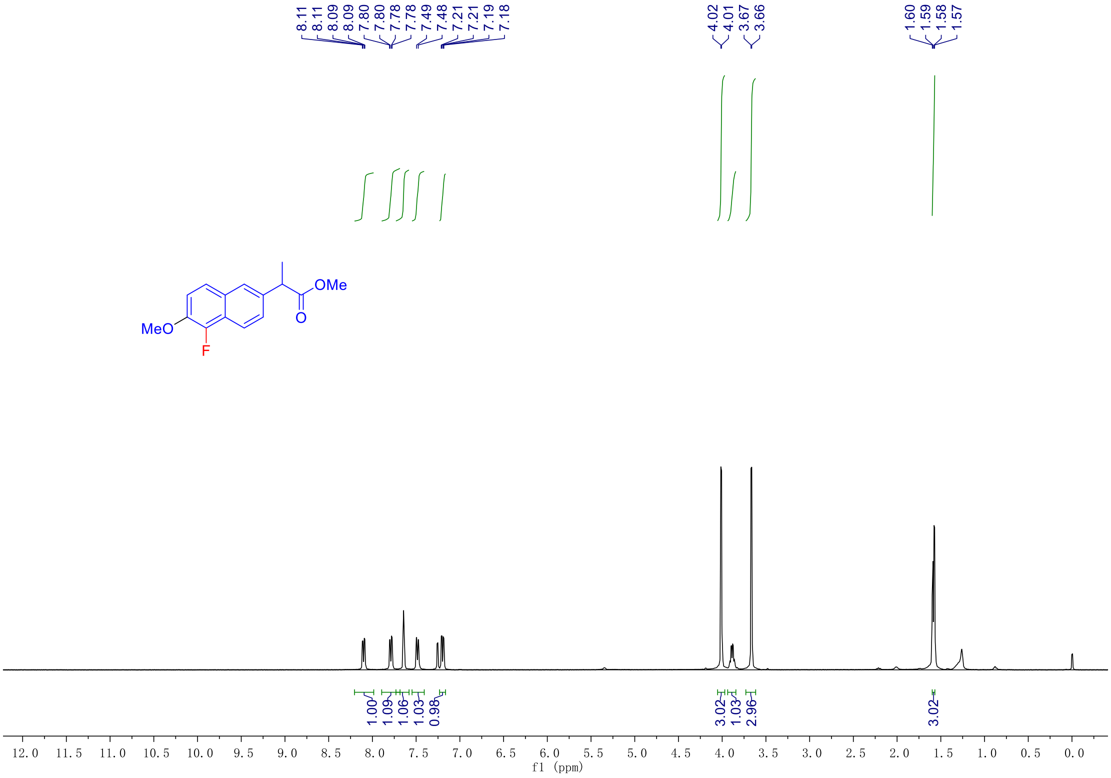

Figure S16. ^{1}H-NMR Spectrum (400 MHz, $CDCl_3$) of **4a.**

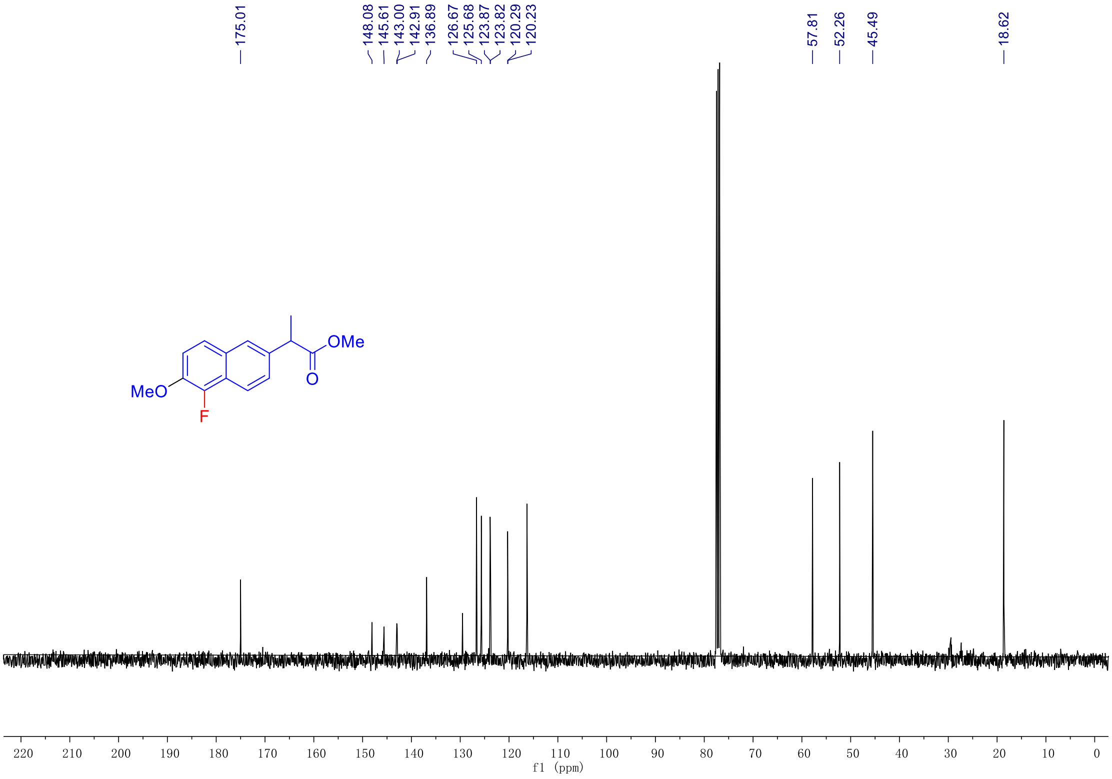

Figure S17. ^{13}C-NMR Spectrum (101 MHz, $CDCl_3$) of **4a.**

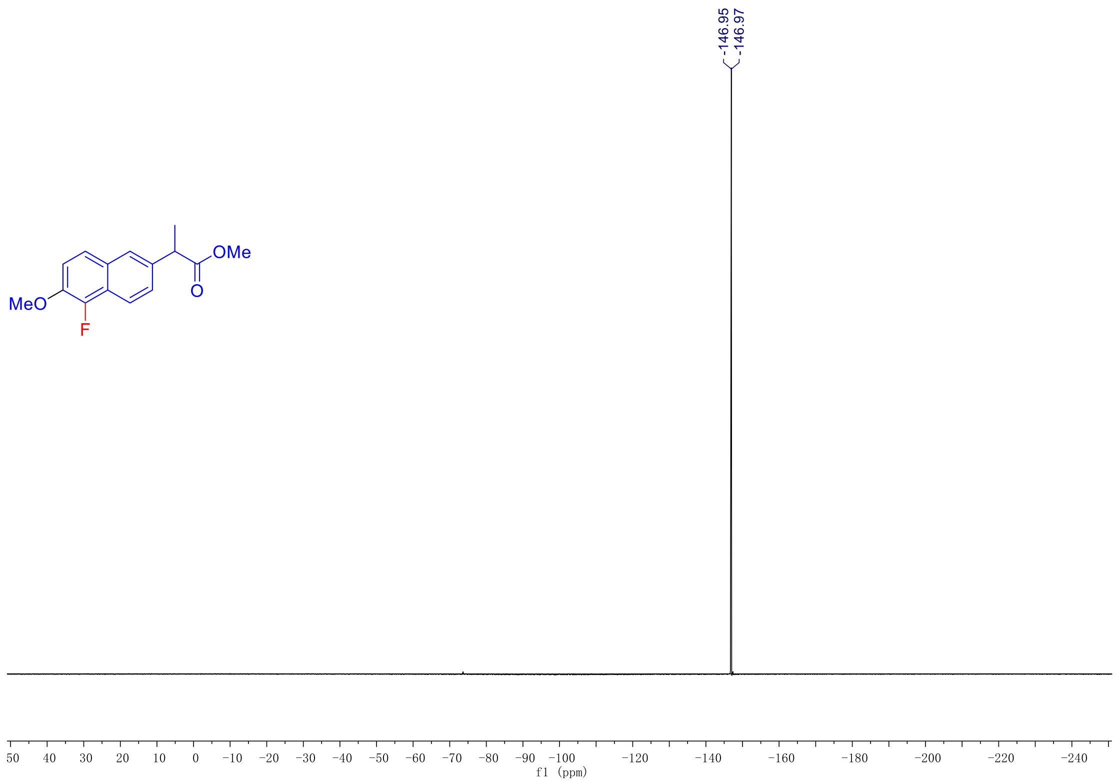

Figure S18. ^{19}F-NMR Spectrum (376 MHz, CDCl$_3$) of **4a.**

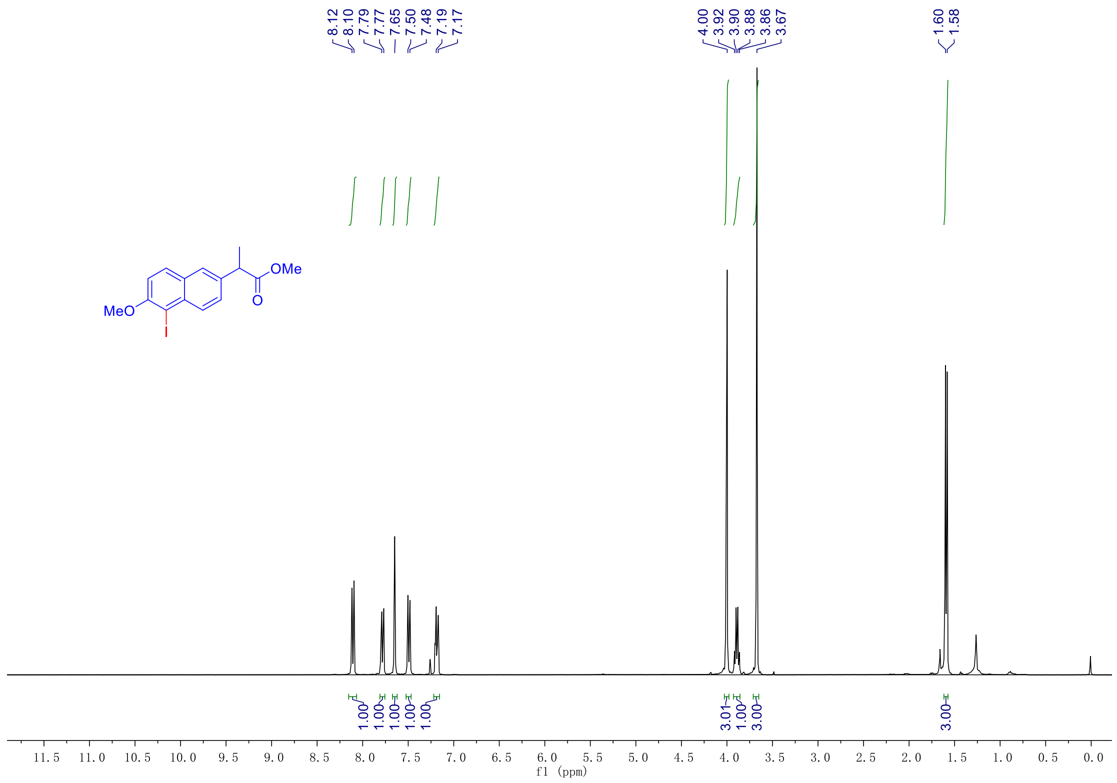

Figure S19. ^{1}H-NMR Spectrum (400 MHz, CDCl$_3$) of **4b.**

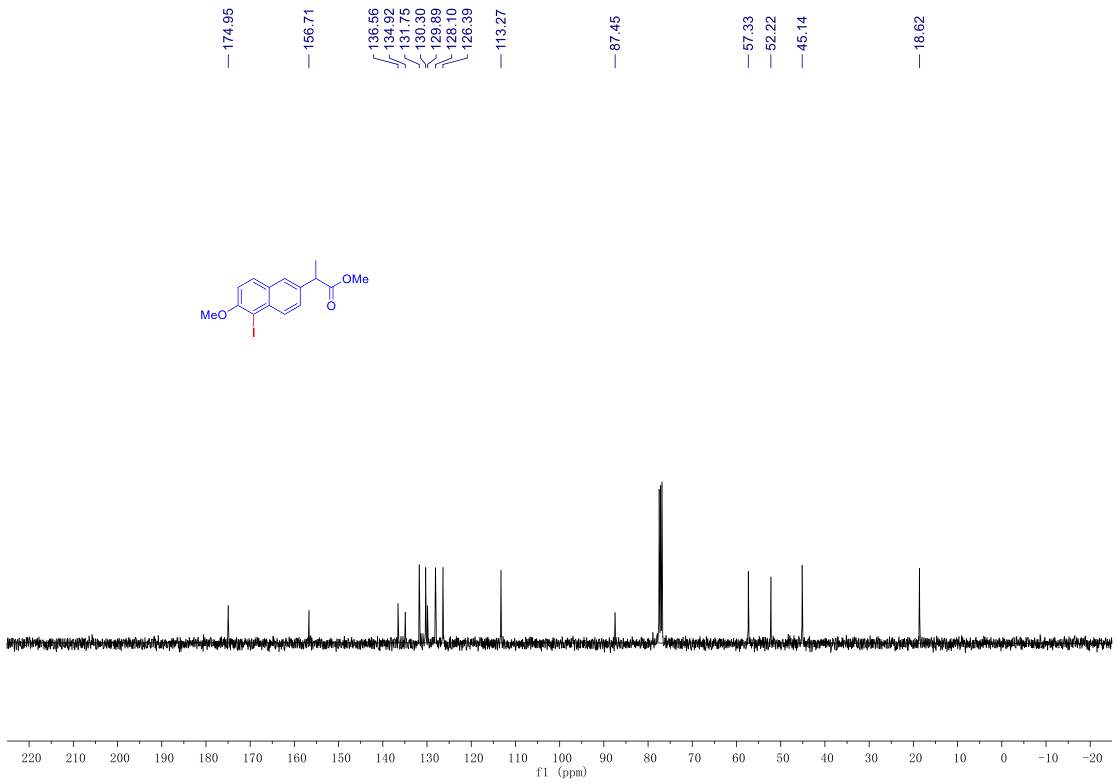

Figure S20. ^{13}C-NMR Spectrum (101 MHz, $CDCl_3$) of **4b.**

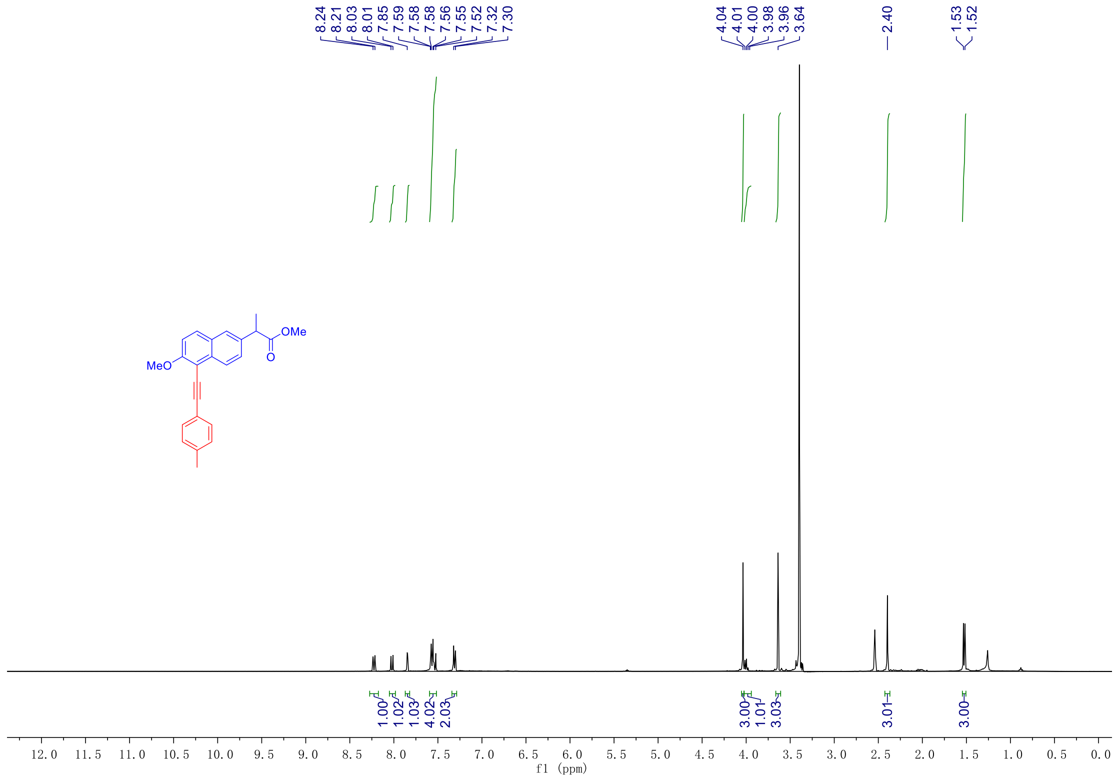

Figure S21. ^{1}H-NMR Spectrum (400 MHz, DMSO-d6) of **4c.**

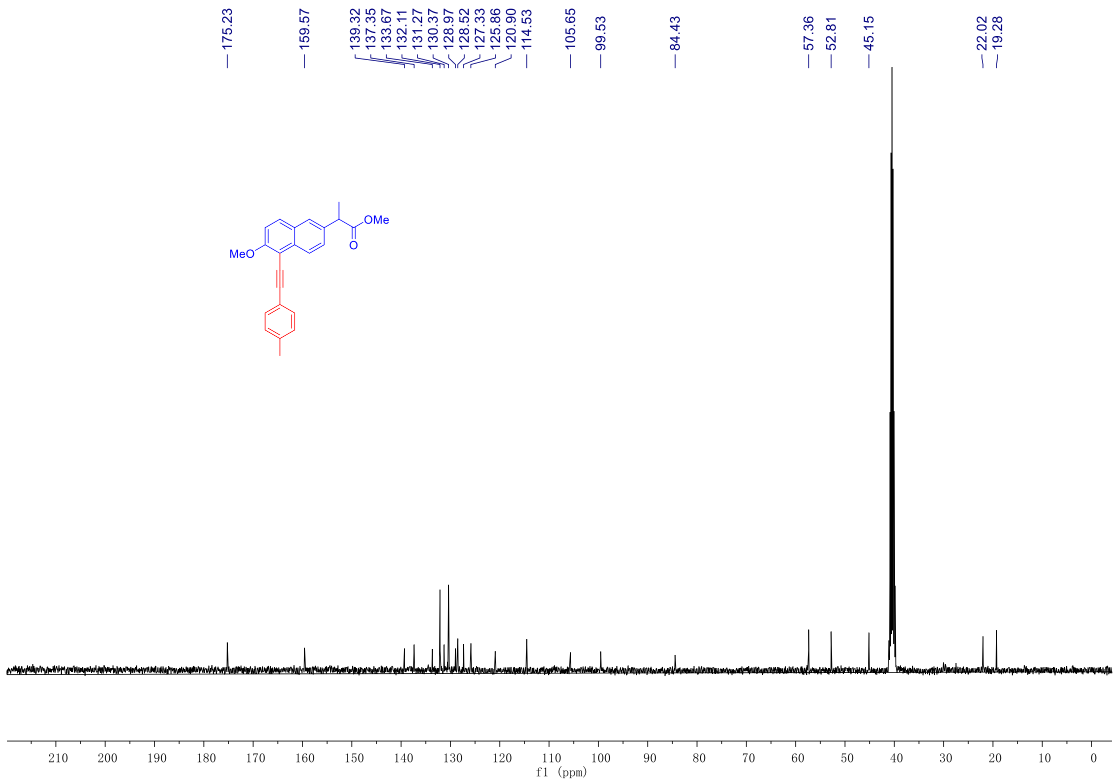

Figure S22. ^{13}C-NMR Spectrum (101 MHz, DMSO-d6) of **4c.**

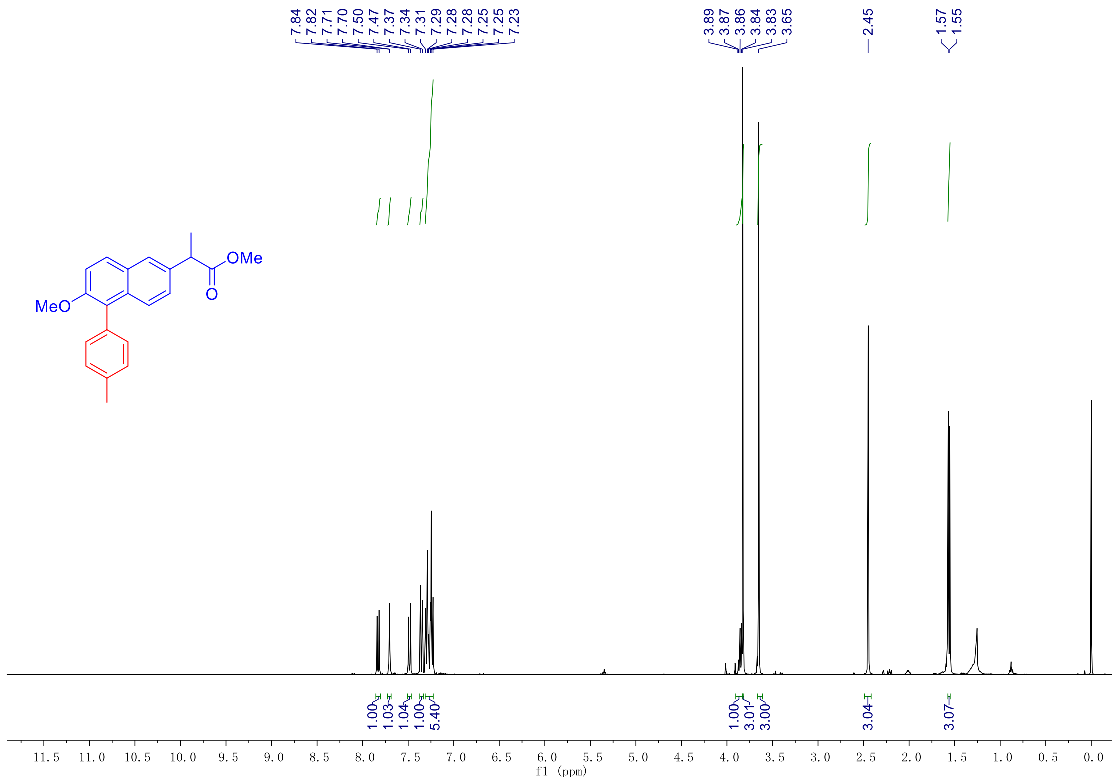

Figure S23. ^{1}H-NMR Spectrum (400 MHz, $CDCl_3$) of **4d.**

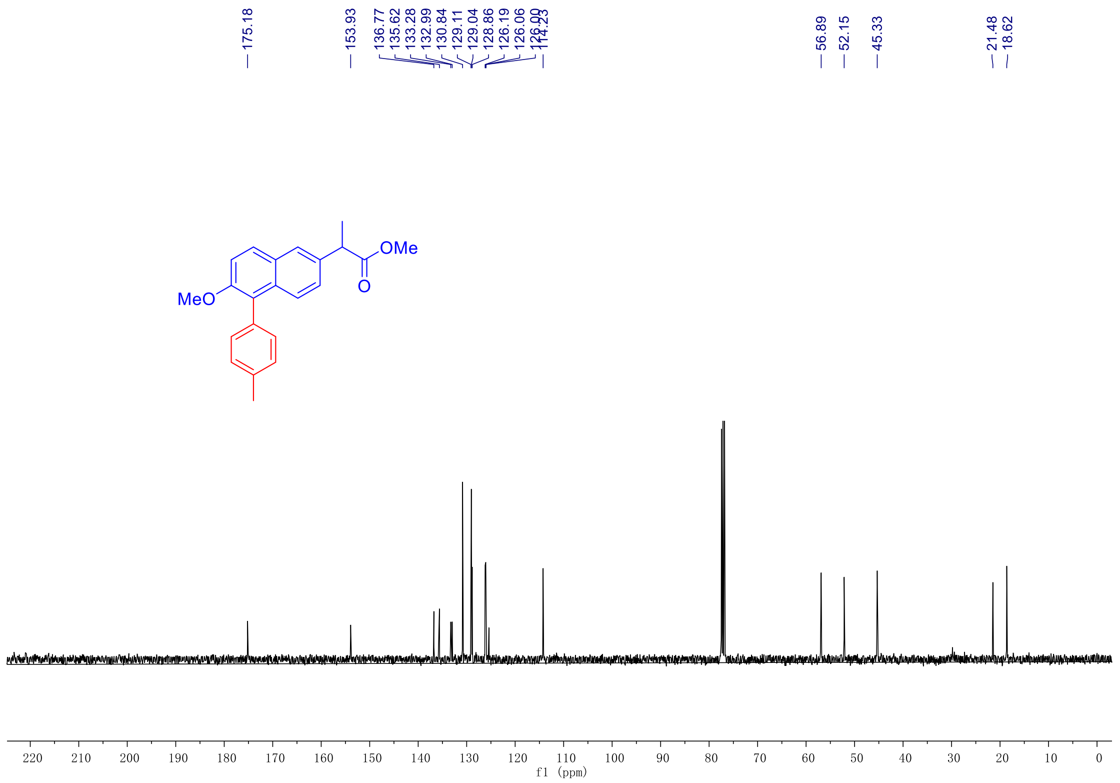

Figure 24. ^{13}C-NMR Spectrum (101 MHz, $CDCl_3$) of **4d.**

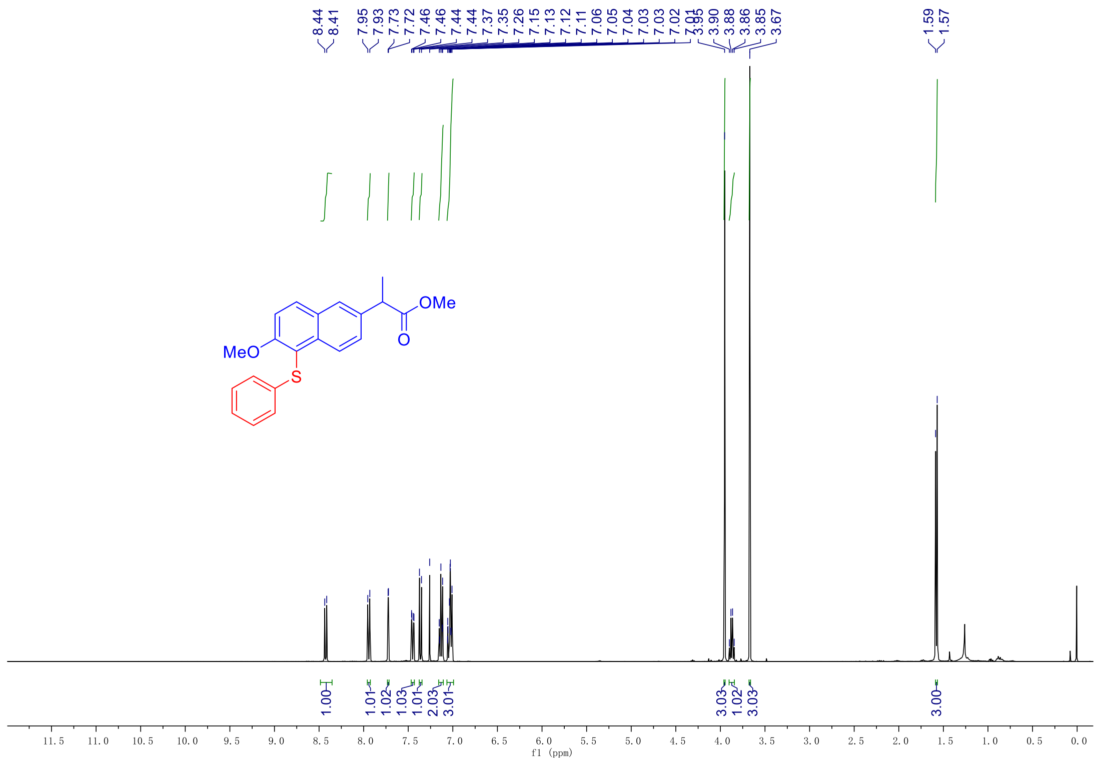

Figure S25. ^{1}H-NMR Spectrum (400 MHz, $CDCl_3$) of **4e.**

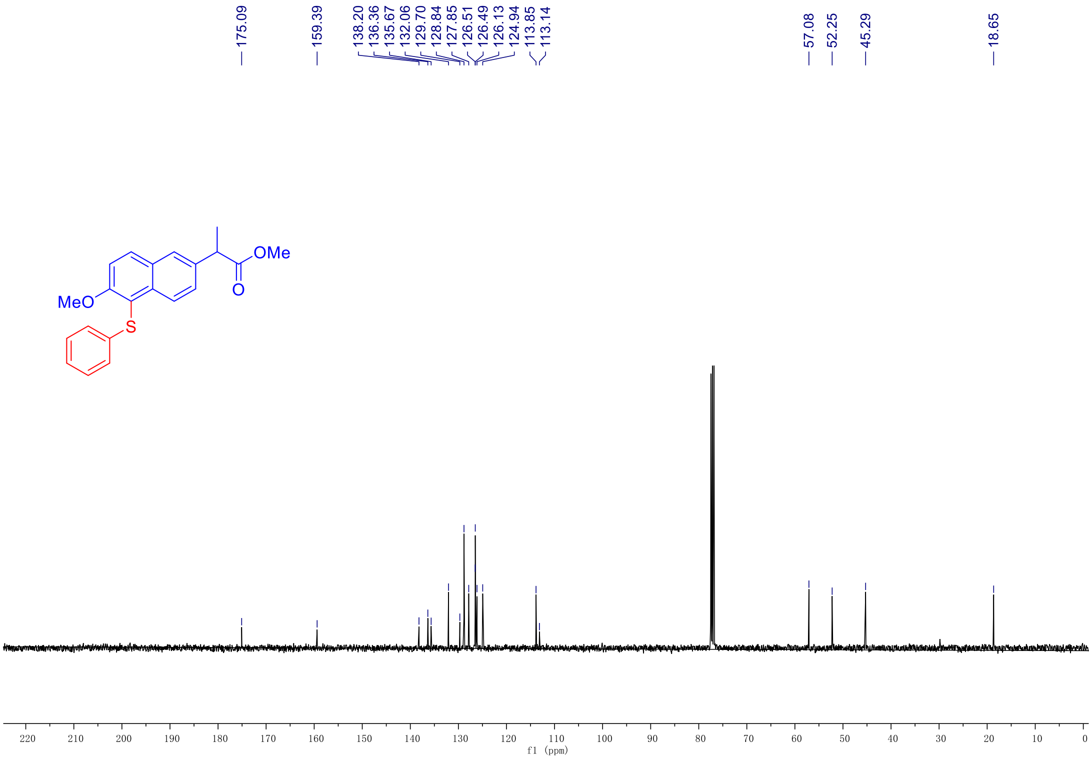

Figure S26. ^{13}C-NMR Spectrum (101 MHz, $CDCl_3$) of **4e.**

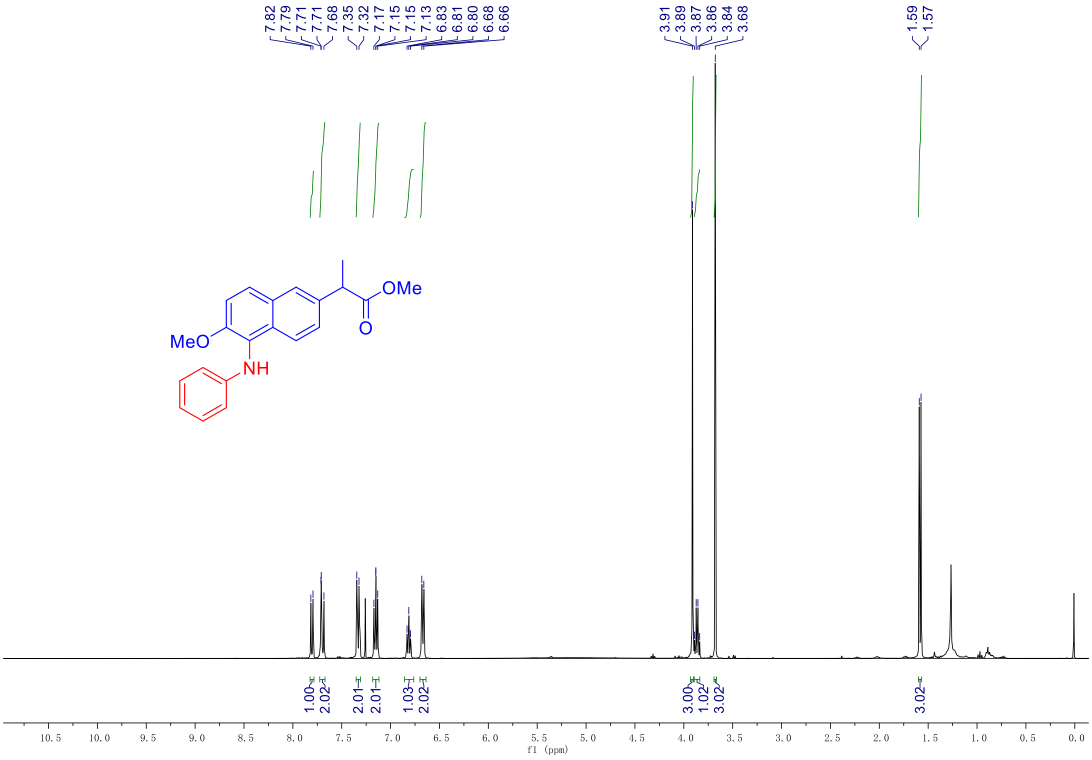

Figure 27. ^{1}H-NMR Spectrum (400 MHz, CDCl3) of **4f.**

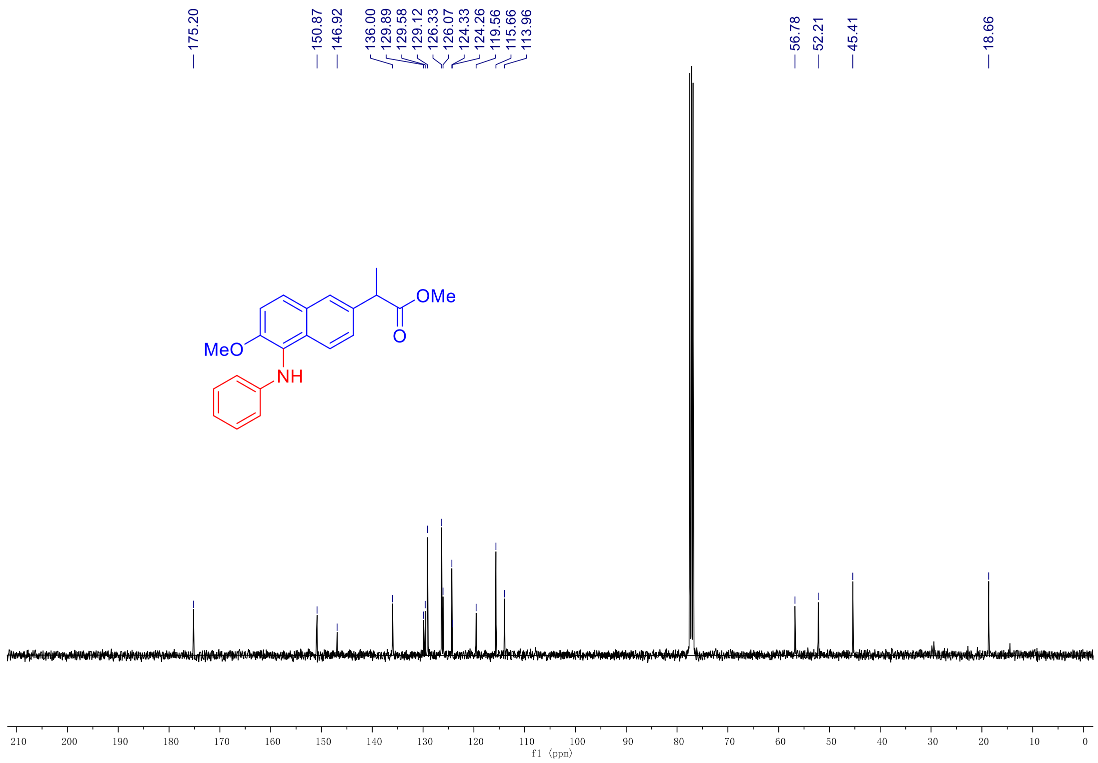

Figure S28. ^{13}C-NMR Spectrum (101 MHz, $CDCl_3$) of **4f.**

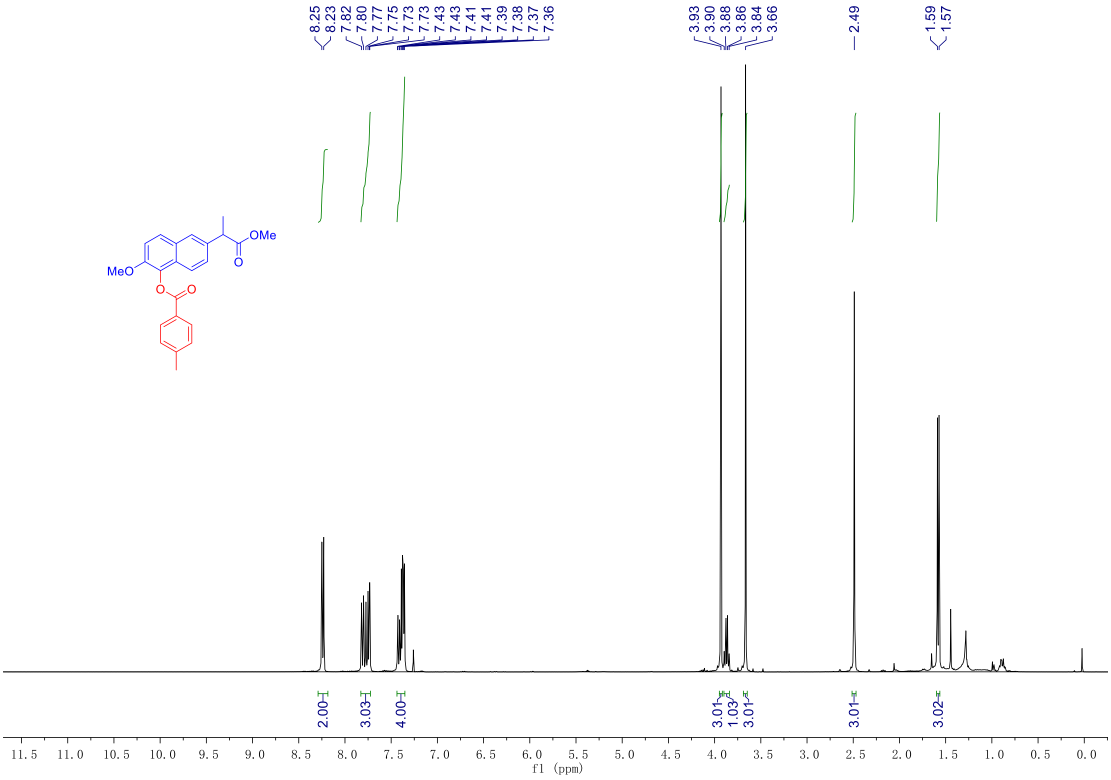

Figure S29. ^{1}H-NMR Spectrum (400 MHz, $CDCl_3$) of **4g.**

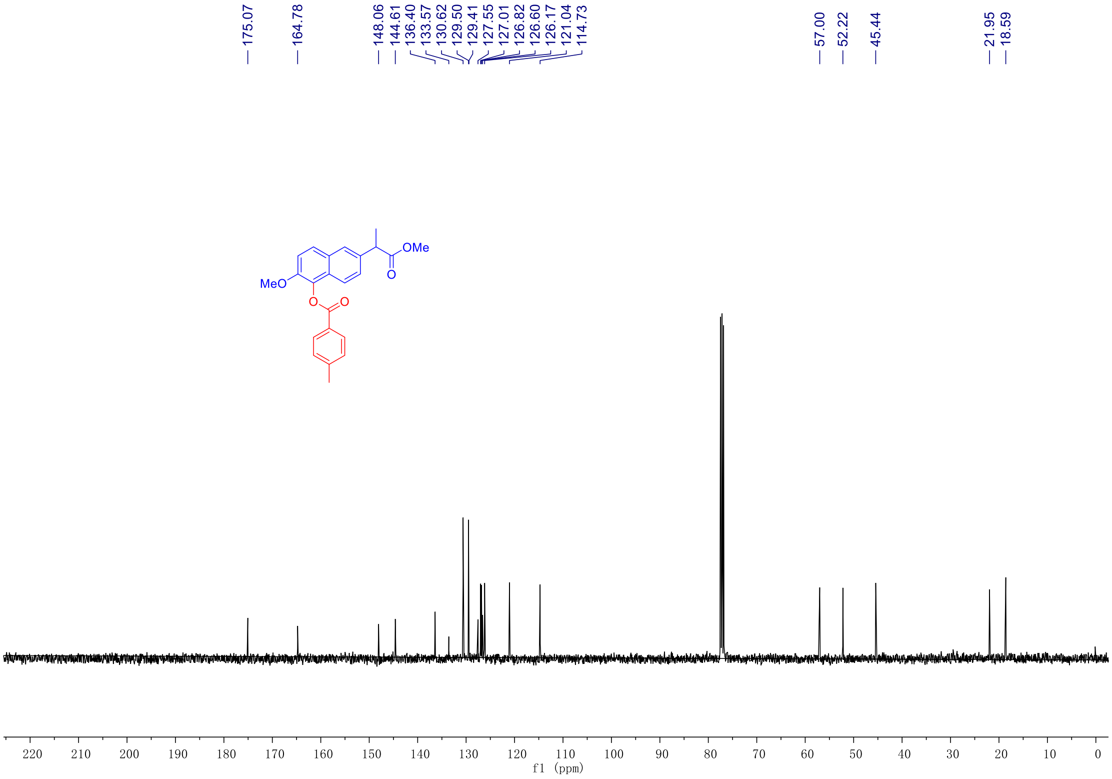

Figure S30. ^{13}C-NMR Spectrum (101 MHz, $CDCl_3$) of **4g.**

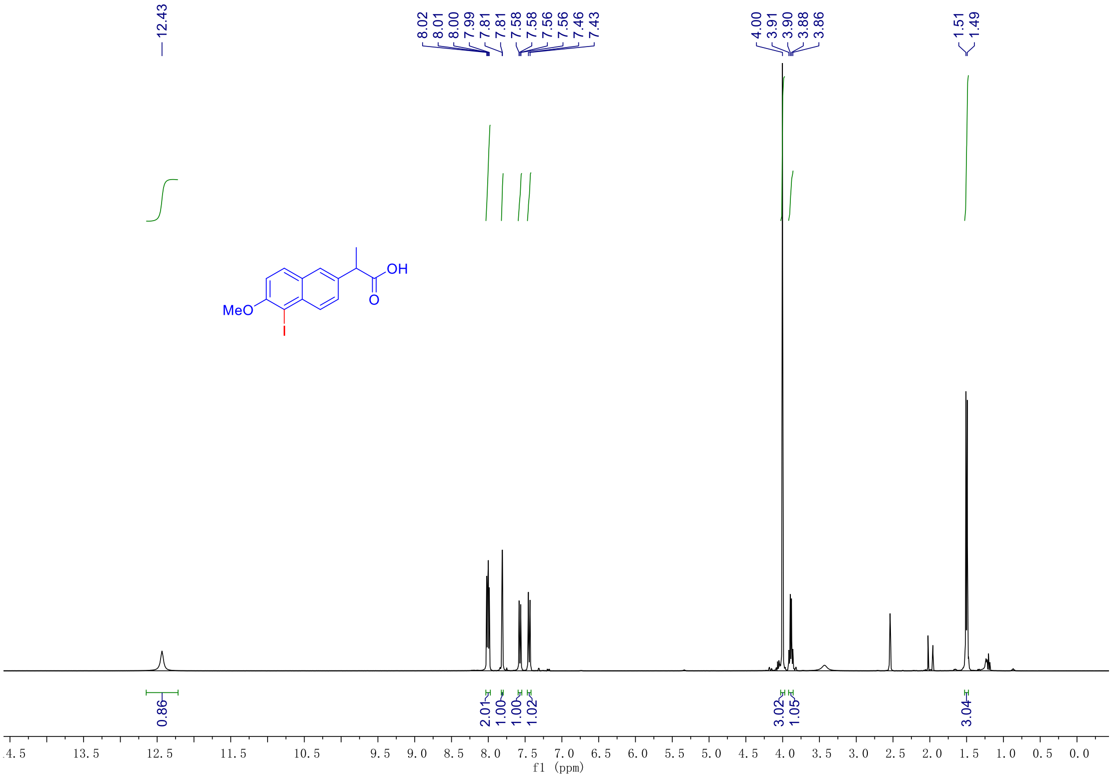

Figure S31. ^{1}H-NMR Spectrum (400 MHz, DMSO-d6) of **6.**

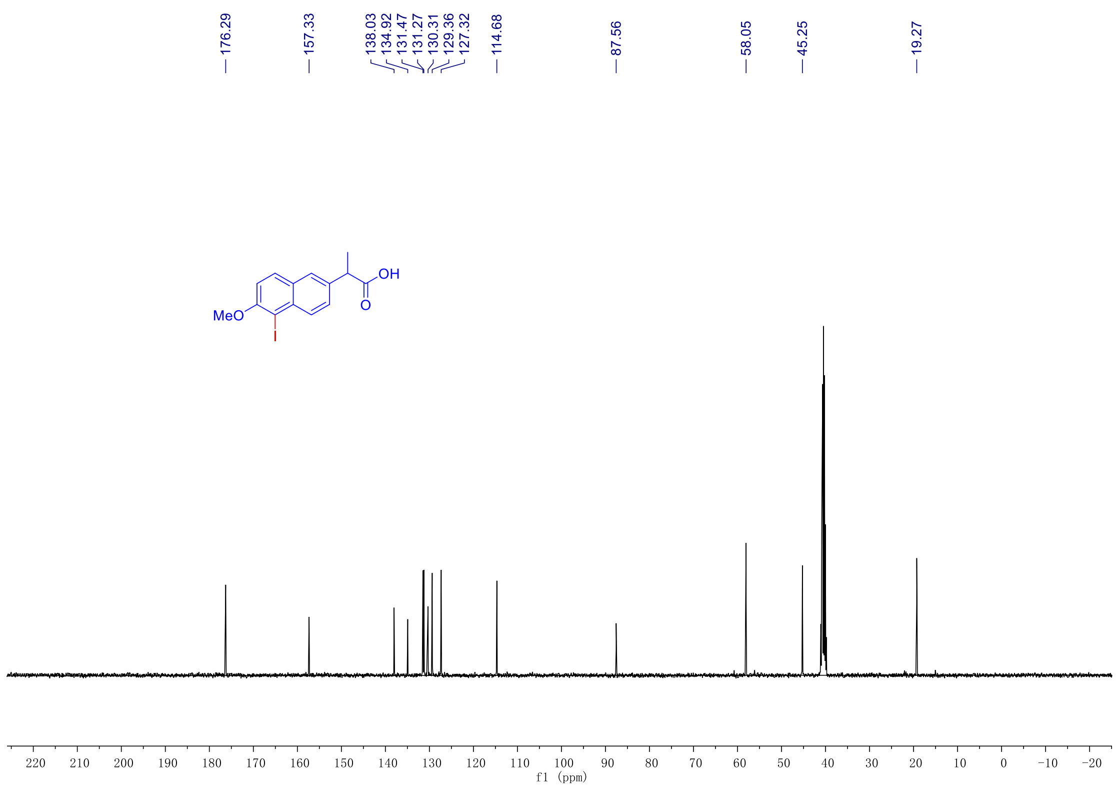

Figure S32. ^{13}C-NMR Spectrum (101 MHz, DMSO-d6) of **6.**
